# Functional connectivity of task context representations in prefrontal nodes of the multiple demand network

**DOI:** 10.1007/s00429-018-1638-9

**Published:** 2018-03-03

**Authors:** Peter Stiers, Alexandros Goulas

**Affiliations:** 1 Department of Neuropsychology and Psychopharmacology, Maastricht University, Universiteitssingel 40 (East), 6229 ER Maastricht, The Netherlands; 2 Department of Computational Neuroscience, University Medical Center Hamburg-Eppendorf, Martinistr. 52, 20246 Hamburg, Germany

**Keywords:** Cerebral cortex organisation, Inferior frontal junction, PreSMA, Resting state functional connectivity, Task-related fMRI, Anterior insula

## Abstract

A subset of regions in the lateral and medial prefrontal cortex and the anterior insula increase their activity level whenever a cognitive task becomes more demanding, regardless of the specific nature of this demand. During execution of a task, these areas and the surrounding cortex temporally encode aspects of the task context in spatially distributed patterns of activity. It is not clear whether these patterns reflect underlying anatomical subnetworks that still exist when task execution has finished. We use fMRI in 12 participants performing alternating blocks of three cognitive tasks to address this question. A first data set is used to define multiple demand regions in each participant. A second dataset from the same participants is used to determine multiple demand voxel assemblies with a preference for one task over the others. We then show that these voxels remain functionally coupled during execution of non-preferred tasks and that they exhibit stronger functional connectivity during rest. This indicates that the assemblies of task preference sharing voxels reflect patterns of underlying anatomical connections. Moreover, we show that voxels preferring the same task have more similar whole brain functional connectivity profiles that are consistent across participants. This suggests that voxel assemblies differ in patterns of input–output connections, most likely reflecting task demand-specific information exchange.

**Electronic supplementary material:**

The online version of this article (10.1007/s00429-018-1638-9) contains supplementary material, which is available to authorized users.

## Introduction

The ability to select in each situation the most appropriate action, given the available options and the learned regularities of that situation, is largely depending on a network of brain areas in the parietal and prefrontal lobes (Goldman-Rakic 1988; Miller and Cohen 2001; Corbetta and Shulman 2002; Duncan 2013). Particularly, the areas around the inferior frontal sulcus have been associated with the maintaining of task rules (Bengtsson et al. 2009; Wallis et al. 2001; White and Wise 1999), whereas the superior parietal regions are thought to be involved in tracking the available environmental cues in task relevant priority maps (Bisley, and Goldberg 2010; Womelsdorf and Everling 2015).

Areas in the lateral and the medial posterior prefrontal cortex, together with the anterior insula, have been shown to be active during a wide range of tasks (Duncan and Owen 2000; Mennes et al. 2006). Moreover, they have been shown to modulate their level of activation with cognitive effort, regardless of the specific demands of the task (Duncan 2010; Stiers et al. 2010). Thus, the neural activity in these areas increases when the number of elements in a working memory task increases, but also when the stakes in a gambling task become more risky. This has been referred to as their “multiple demand property” (Duncan 2010, 2013).

The neural mechanism behind this multiple demand property is not yet clear. The property may arise because these areas have the same functional contribution in each of these tasks. However, increasing evidence suggests that areas in the prefrontal cortex represent different aspects of the task context in a unique, spatially distributed pattern of neuronal activity. Thus, activity patterns in the cortical region covering the inferior frontal sulcus allow decoding specific aspects of the task context, such as what stimulus feature a person attends to, or the specific response mapping rule that s/he follows (Woolgar et al. 2011; Waskom et al. 2014). Moreover, physiological recordings in animals have shown that the pattern of neural activity in this area changes with the task being performed or the particular phase of a task the subject is in (Saga et al. 2011; Sigala et al. 2008; Kaping et al. 2011). These results suggest that even though different tasks significantly activate the same region of cortex, specific tasks induce a unique pattern of activity. This is confirmed by the fMRI finding that subsets of voxels distributed throughout the multiple demand nodes show reliable preferences for one or more of a set of tasks (Stiers et al. 2010).

At the neural level, these unique patterns are thought to reflect the activity of different, but spatially intermingled subpopulations of neurons that are engaged during the execution of the different tasks. Physiological recordings of local field potentials suggest that these subpopulations form task context-specific assemblies (Sigala et al. 2008; Voloh et al. 2015). Differences in task preferences of fMRI voxels are then thought to reflect differences in densities of task context tuned cell assemblies within these individual voxels (Haynes and Rees 2005; Kamitani and Tong 2005; Norman et al. 2006; But, see Gardumi et al. (2016) for a discussion of alternative views). The neurons across network nodes that constitute assemblies during a particular task context are assumed to be interconnected and to exchange information relevant for the execution of their preferred task. This assumption receives support from fMRI data, showing that multiple demand voxels preferring the same task have a stronger functional coupling during execution of that task than voxels that differ in preference (Stiers et al. 2010). This is not only true for voxels within a particular node, but also for voxels located in different nodes of the network.

In the present study, we further elaborate on this model of the intrinsic organisation of the multiple demand network. A question of particular interest is the temporal flexibility of neuron assemblies. Within the time frame of on-going performance, the flexibility of neural assemblies has been convincingly demonstrated. Multi-array cell recordings show that assemblies of neurons and their representation contents appear and disappear as the animal transits from one task phase to the next (Saga et al. 2011; Sigala et al. 2008; Stokes et al. 2013). This flexibility is comparable to the emergence and disappearance of activation patterns in early sensory areas contingent upon receptor stimulations. The rapid transitions between activity patterns in PFC are thought to rely on a special form of “dynamic” coding in large arrays of neurons (Duncan 2001; Riggotti et al. 2010; Stokes et al. 2017). These arrays allow the formation of functional episodes (Duncan 2013) or (effective) connectivity states (Stokes et al. 2017) that variably bind combinations of sensory, motivational and motor contexts relevant for the current task. An unanswered question is what happens to assemblies after activity in their circuits has disappeared because the person transited to a new task phase. On one end of the theoretical continuum, the neurons in the array exhibit equipotentiality for representing information that is relevant for the task. The array is wired in such a way that it can temporally represent any task context for the time needed, much in the same way that a sensory input array temporarily represents a particular sensory input pattern. As is the case for sensory neurons, representational equipotentiality translates into unbiased interconnections between the dynamic coding neurons. On the other end of the continuum, there is the view that the task context specific assemblies are established during acquisition of the specific tasks and rely on hard-wired connections between the neurons, which still exist when not engaged in the specific task.^1^ A first indication that task context specific patterns of activation are stably formed was provided in Stiers et al. (2010). They found stronger functional coupling between multiple demand voxels tuned to the same task than voxels with non-matched preferences, even when the task executed was not the task to which the voxels were tuned. A further step in demonstrating the stability of task context-specific assemblies was recently provided by Waskom and Wagner (2017). They showed that voxels in the larger inferior frontal sulcus cortex that contributed to decoding attention to a particular stimulus dimension (i.e. shape, colour or structure) showed stronger functional coupling also outside of the task, i.e. during rest. These findings suggest that the specific prefrontal paths of neuronal information exchange underlying performance in different tasks do not cease to exist upon task termination, but are still present at a later time point.

In the present study, we investigated whether stable functional coupling, i.e. beyond the time window of task execution, also exists between multiple demand voxels that share a tuning for a specific task across nodes of the multiple demand network. To identify task preference sharing voxels, we administered short blocks of three different tasks to 12 participants while in the scanner. Multiple demand nodes were identified by isolating voxels that were activated by all three tasks and by showing that their activity was modulated by task difficulty in all three tasks. Based on our previous work (Stiers et al. 2010), four different multiple demand nodes were selected (see Table 1) in each participant, and the node voxels were sorted based on their preference profile for the three tasks. This allowed us to study functional couplings between classes of voxels sharing the same preference profile. We studied functional coupling strength during task execution and during independently acquired fMRI data during rest, and compared same and differently tuned voxels within nodes and between nodes. The analyses show evidence of stronger coupling of voxels, forming an interregional task-specific assembly, in all measures. These results could be expected given neuroanatomical reports of discrete groups of neurons, organized in stripe-like patterns that form subnetworks across the areas of the association cortex (Pucak et al. 1996; Selemon and Goldman-Rakic 1988; Marconi et al. 2001). Such a structural skeleton might be the basis of the functional subnetworks evidenced in our fMRI measurements.

**Table 1:** Regions of interest location

Activation sites		*N* of subjects^a^		MNI coordinates						*N* voxels		Anatomy
				*x* (mm)		*y* (mm)		*z* (mm)				
Name	Side	Bilat	Unilat	*M*	SD	*M*	SD	*M*	SD	*M*	SD	
IFS1	Left	12	12	− 50.2	7.4	3.8	3.9	35.7	6.3	170.3	49.3	Inferior frontal junction
	Right	12	12	50.8	6.5	6.3	3.6	34.3	3.6	203.8	44.8	
IFS2	Left	9	10	− 47.3	8.5	18.0	4.6	26.2	2.1	58.3	44.3	Inferior frontal sulcus
	Right	9	10	51.6	3.8	18.9	5.1	28.7	6.9	63.9	71.1	
INSa	Left	12	12	− 33.8	4.2	20.1	4.8	4.2	4.7	70.1	56.9	Anterior bend of circular sulcus
	Right	12	12	37.8	4.3	18.7	4.3	3.0	2.3	88.9	47.9	
MSFG	Left	12	12	− 8.3	1.9	7.3	4.4	53.8	4.5	257.3	95.9	Superior frontal gyrus (preSMA) and cingulate sulcus (RCZ)
	Right	12	12	8.8	2.0	8.6	4.7	57.0	3.4	255.8	97.1	

*IFS* inferior frontal sulcus, *INSa* anterior insula, *MSFG* medial superior frontal gyrus, *preSMA* presupplementary motor area, *RCZ* rostral cingulate motor zone, *MNI* Montreal Neurological Institute

^a^Number of individual data sets in which the shared activation side could be identified, either bilateral or unilateral

## Methods

### Participants

Twelve people participated voluntarily in this study. Their age ranged from 20 to 47 years and 7 were females. They were right handed and free of any neurological or psychiatric conditions. All participants gave their informed consent to participate in the study, which was approved by the local ethics committee.

### Behavioural tasks

**Fig. 1:**
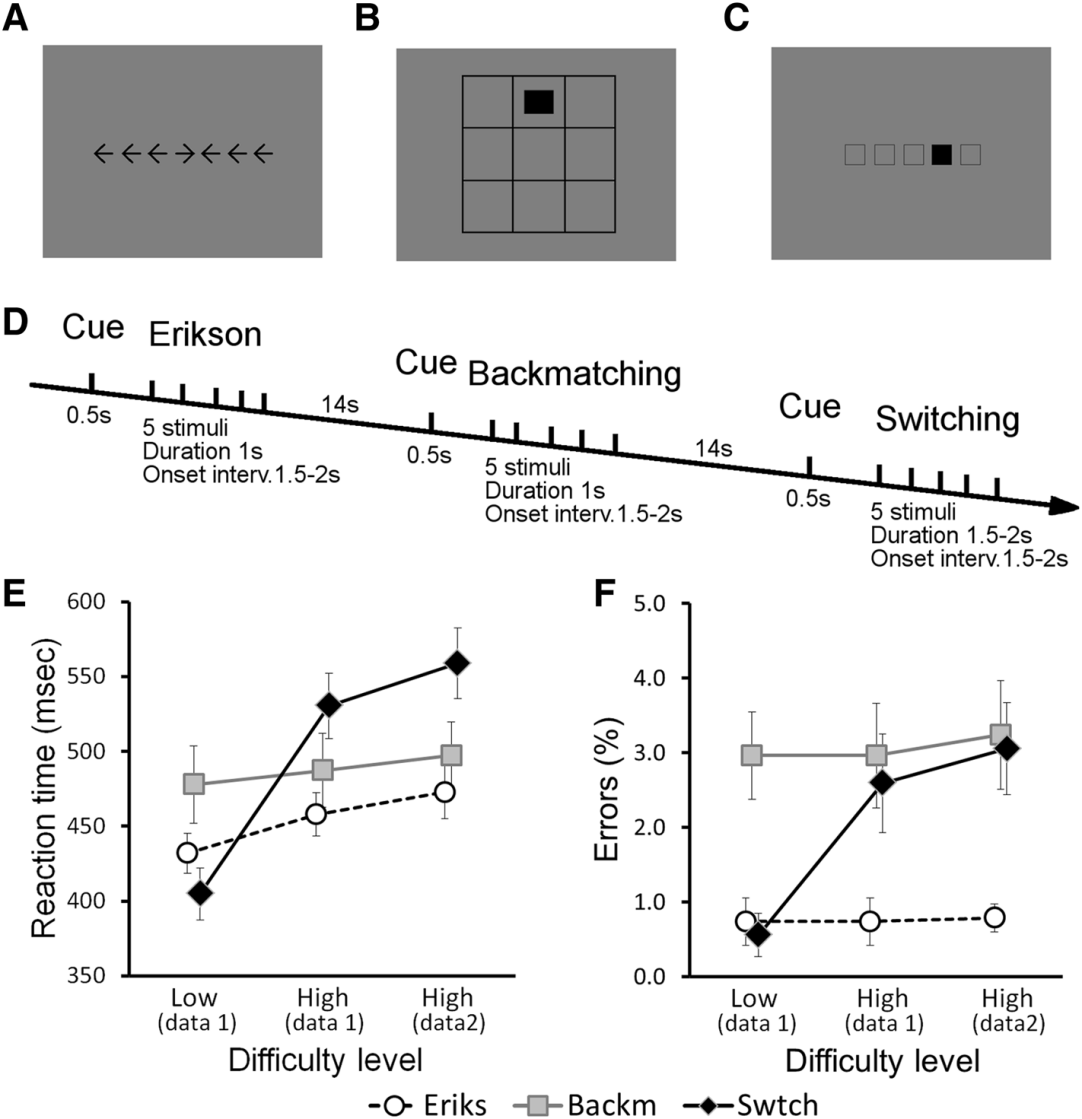
Behavioural paradigm. **a**–**c** Illustration of the type of stimuli used in each of the three tasks: **a** Eriksen flanker task; **b** Backmatching; **c** response scheme switching. **d** Temporal organization of the paradigm. The three tasks were administered alternatively in block of five trials per task with an inter-block interval of 14 s. Each block was initiated by a cue stimulus indicating which task participants were going to perform. See text for more detailed description of the tasks. **e**–**f** Summary of behavioural performance. Reaction time (**e**) and percentage of error trials (**f**) in the low and high difficulty conditions of each of the three tasks. Data set 1 consisted of blocks of easy and difficult trials for each task, whereas data set 2 only had blocks of difficult trials. Error bars indicate ± 1 SE. Error trials were excluded from these behavioural analyses as well as from the fMRI analyses where they were modelled as separate events of no interest

Three different cognitive tasks were administered in each imaging run. The task paradigms were created and administered using Presentation software (Neurobehavioral Systems, Albany, CA, USA). The stimuli used are illustrated in Fig. 1a–c. They consisted of black and white patterns set at maximum contrast against an intermediate grey background [RGB settings (1, 1, 1), (255, 255, 255), and (127, 127, 127), respectively]. The stimuli were optically back-projected from the technical room of the scanner onto a semi-transparent plastic screen 17 cm high and 25 cm wide positioned vertically against the upper internal surface of the scanner bore. Participants saw the reflections of the stimuli in a mirror fixed at the top of the head coil at 29 cm from the screen and ± 6 cm from the participants’ eyes. The stimuli comprised a visual angle of ± 15.5° (9.5 cm) horizontally.

The tasks were administered in blocks of five consecutive trials of the same task. The tasks were alternated in a pseudo-random order with the restriction that no task was administered in two consecutive blocks. The time between blocks was 14 s (Fig. 1d). One run consisted of 6 blocks of each task for a total of 18 blocks. Two types of runs were created. In the first type, half of the blocks of each task contained easy trials, requiring less cognitive effort, while the other half included difficult trials. Three such runs were administered to the participants in one scanning session. Data from these runs were used to identify multiple demand areas. In the second type of runs, all six blocks of each task contained difficult trials. Six runs of this type were administered to each participant in a separate scanning session and these data were used to study voxel preference profiles and functional couplings during task execution.

What task would be administered in each block was indicated by a cue prior to the start of the first trial. This cue was visible for 500 msec and consisted of the first letter of the task (“E” for Eriksen, “B” for Backmatching, etc.). The colour of the letter indicated the difficulty class of the block, with white letters indicating easy blocks and black letters indicating difficult blocks. The first trial in each block started 4 s after the onset of the cue. For subsequent trials, the trial onset interval varied randomly between 1.5 and 2.0 s, in 100 ms steps. For the Eriksen and Backmatching tasks, 1000 ms of this interval was occupied by the presentation of the stimulus. For Switching, the stimulus remained visible for the entire interval to avoid working memory demands.

In each trail, participants had to make their choice known via a standard MRI-compatible key pad, which was operated by the index and middle finger of the right hand. For direction responses in the Eriksen and the Switching task, left and right directions were indicated by the index and middle finger, respectively. For match/non-match responses in the Backmatching task, the index finger indicated a match and the middle finger a mismatch. Hence, a motor response was required in all trials. A description of the stimulus material and difficulty level for each task is provided below:

*Eriksen task* (Fig. 1a) In each trial, participants had to indicate the left or right direction in which a centrally positioned arrow head was pointing. The arrow head was flanked on the left and the right with similar arrow heads that pointed either in the same (congruent) or the opposite direction (incongruent) as the central arrow head. In the “easy” block, all five trials were congruent, whereas difficult blocks consisted of a random alternation between two congruent and three incongruent stimuli.

*Backmatching task* (Fig. 1b) The spatial lay-out of the stimuli in this spatial Backmatching task was a three-by-three array of empty squares. A stimulus consisted of the appearance of the letter “X” centrally in one of the nine squares for 1 s. Participants had to match the position of the X in the current trial with its position in previous trials and indicate by key press whether the positions matched. In the “easy” blocks, all trials were 1-back, meaning that the participant had to match stimulus position between the current and the just preceding trial. In the “difficult” blocks, all trials were 2-back, requiring a key press whenever the current stimulus position was identical to its position two trials back in time. Because information needs to be retained over trials, the first trial in a 1-back and the first two trials in a 2-back block could not be a target. Therefore, targets could only appear in the last three trials of a difficult block. Nonetheless, participants needed to start monitoring and feeding information into their working memory starting with the first trial.

*Response scheme switching task* (Fig. 1c) In the last task, the stimulus array consisted of five potential stimulus positions horizontally arranged and marked with five open squares. A stimulus consisted of a full square that filled one of the place holders. Participants had to indicate with a left or right hand key press whether the square in the current trial had jumped to the left or to the right relative to its position on the preceding trial. In the easy trials, the stimulus square was black, and in that case they had to press the key on the side towards which the square had jumped (congruent trial). In difficulty trials, the stimulus square turned white, and in that case the stimulus response mapping rule reversed and participants had to press the key opposite to the direction of the jump (incongruent trial). In easy blocks, the colour of the stimulus square remained black throughout the five trials, whereas in difficult blocks the colour of the stimulus square turned white on two of the five occasions. The first stimulus in a block was always black and did not require a response from the participants.

Although difficult blocks of the Eriksen and the Switching task contained both easy and difficult trials, all trials in a difficult block will be called “difficult trials”, because the mixed organisation of trials within these blocks increases the cognitive effort of the block as a whole. The average reaction times of individual participants in each of the tasks and difficulty levels were subjected to a repeated measures analysis of variance with three task types (Eriksen, Backmatching, Switching) and three difficulty level of the trials (Easy-data set 1, Difficult-data set 1, Difficult-data set 2). Greenhouse-Geisser correction was applied to compensate for deviations of sphericity. Follow-up analyses comprised paired t tests, with Bonferroni correction for multiple comparisons.

### MRI image processing

Data were collected on a Siemens MAGNETOM Allegra 3T MRI head-only scanner. Head motion was constrained by the use of foam padding. All functional images were acquired with a T2*-weighted gradient echo planner pulse sequence covering the whole brain including the cerebellum with 32 axial slices (TR 2000 ms, TE 30 ms, FA 90°, FOV 224, slice thickness 4 mm, matrix size 64 × 64, flip angle 90°). Voxel size was 3.5 × 3.5 × 4 mm. A gradient echo image (TR 704 ms, TE 5.11 and 7.57 ms; flip angle 60°) with the same grid and slice orientation as the functional images was acquired to generate a field map for correcting susceptibility-related distortions in the functional images. A T1-weighted anatomical scan was also acquired (TR 2250 ms, TE 2.6 ms, flip angle 9°, FOV 256 mm, slice thickness 1 mm, matrix size 256 × 256, number of slices 192). Voxel size was 1 × 1 × 1 mm.

#### Preprocessing

The data were pre-processed using the SPM5 software (Wellcome Department of Cognitive Neurology, University College, London, UK). Functional volumes were re-aligned, spatially corrected using the field map, slice time corrected, and co-registered with the anatomical scan. The individual T1-weighted anatomical scan was segmented into tissue density maps (grey matter, white matter and CSF) and non-linearly normalized to the MNI template in an integrated procedure. The resulting normalization parameters were applied to the functional data of that participant. In the process, the functional volumes were re-sampled to 2 mm isotropic voxels.

#### Handling of data set 1: three runs with task difficulty manipulation

The three runs with easy and more difficult blocks of each task were analysed separately to identify voxels that were activated by all three tasks and to establish their multiple demand property. We will refer to these data as data set 1. These data were smoothed with a 6 mm FWHM Gaussian kernel prior to entering first level statistical analysis.

In the first level analysis, individual time fluctuations in the BOLD signal were modelled with variables marking the occurrence of individual trials. For each task, the easy and difficult trials were modelled as separate events. Error trials were modelled separately as variables of no interest, as were the task cues preceding each block of trials. Each of these variables was convoluted with the theoretical hemodynamic response function and its time and dispersion derivatives. Session-specific mean regressors were added to neutralize baseline signal differences per run. Lastly, the six realignment variables were included in the model. After estimation of the weights associated with each of the components in the model, contrasts were defined to estimate the strength of the BOLD response in each difficulty level and task type relative to baseline. The resulting weight maps were used to compute whole brain percent signal change maps, which were used in the follow-up region of interest-based group level analysis to confirm the multiple demand property of voxels selected for further analysis (see “Methods”, subsection “Regions of interest identification”).

#### Handling of data set 2: six runs without task difficulty manipulation

The six runs with only difficult blocks of each task were used as an independent data set to study task preference profiles of and functional couplings between the voxels selected based on data set 1. These data will be referred to as data set 2. No smoothing was applied to data set 2.

Analysis of the data was again at the level of individual participants. The modelling of task-induced BOLD fluctuations was similar to that in data set 1, including the modelling of error trials and task preceding cues as events of no interest, but now all trials were of the difficult type. Again, for each task a present signal change map was created based on the estimated model weights. These maps were used to quantify task preferences of individual voxels (see “Methods”, subsection “Voxel-wise task preference profiles”). In addition, a disjunctive *F*-contrast was computed that monitored any pairwise difference between the tasks. The resulting *F*-map was used to establish whether the profile of task preferences of individual voxels was statistically significant (see “Methods”, subsection “Voxel-wise task preference profiles”). The statistical criterion applied was 0.05, false-discovery rate corrected for multiple comparisons (Genovese et al. 2002).

#### Handling of data set 3: resting state fMRI data

In addition to the task-related fMRI data described above, we also collected 10 min of resting state data with the same epi sequence and imaging parameters. Participants were instructed to fixate a central fixation cross while remaining in a relaxed state, avoiding mentally engaging thoughts. The resting state data were always collected as the first run in a scanning session. In two participants, they were collected 1 and 2.5 months prior to entering this experiment. In 1 participant, they were collected during the first scanning session in which data were collected for the current experiment. In the remaining nine participants, the resting state run was collected at the start of the second scanning session. In these nine participants, the average time interval between the two sessions was 7.1 days. Apart from one participant in which the second session was later on the same day, the minimal interval was 5 days.

The resting state data set was preprocessed as described above. No smoothing was applied. A regression analysis was performed on the data from individual participants to further prepare the data. Linear trends were removed by applying a whitening filter as a standard SPM procedure. Realignment parameters were included as regressors in the analysis model, in addition to the average signal from the white matter and from the CSF. The global brain signal or average grey matter signal was not included as a regressor. Lastly, the session-specific mean and intrinsic autocorrelations were removed from the data, following the standard SPM procedures. Finally, the residual signals of the multiple regression were Fourier band pass filtered from 0.1 to 0.01 Hz to retain only the low-frequency fluctuations in the signal. Only after filtering, volumes contaminated with excessive head movement (volume to volume displacement > 10% of slice thickness, or 0.4 mm) were eliminated.

### Region of interest identification

Regions of interest were identified using data from the three difficulty manipulation runs after smoothing the data with a 6 mm FWHM Gaussian filter. Regions of interest were identified at the level of individual participant data in a GLM conjunction analysis including all three tasks. For each task, a separate t-contrast was constructed that pooled the easy and difficult condition effects. This contrast identified for a particular task the voxels that showed an increased BOLD signal in the easy and/or difficult trials. Next, a conjunction contrast was created involving these three task-specific contrasts to delineate the voxels that showed a significant BOLD response in all three tasks. The statistical significance threshold for this conjunction t-map was FDR corrected for multiple comparisons at the alpha level of 0.05 (Genovese et al. 2002).

The thresholded conjunction maps were used to manually identify the activation clusters corresponding to known commonly activated cognition-related areas in the prefrontal-insular cortex, using anatomical landmarks and spatial coordinates described in Stiers et al. (2010). The following areas were sought for in both hemispheres: on the lateral side at the inferior frontal junction (IFS1), in the inferior frontal sulcus (IFS2), and the anterior insula (INSa), and on the medial side two regions in the middle cingulate cortex (referred to as MF2 and MF3in Stiers et al. 2010). Area MF2, which showed the multiple demand property only on the right side in Stiers et al., was not retained in the analyses for the current study because it was difficult to dissociate from MF3 in the data from many participants. Hence, the area referred to as medial superior frontal gyrus (MSFG) in the current paper may well combine voxels from both MF3 and MF2. Each ROI was defined by a local maximum of BOLD modulation in the conjunction map, and comprised all the voxels within a certain radius of the local maximum that were significant (as defined above) in the conjunction analysis. The radius was selected to reflect the overall activation size at each location and was 6 mm for INSa and IFS2, 8 mm for IFS1 and 10 mm for MSFG.

A ROI-based group analysis was performed on the condition-specific percent signal change (PSC) data derived from the single participant GLM analyses described above, in order to confirm that the activity in these clusters was significantly modulated by task difficulty across the three tasks. This would establish the multiple demand feature of the voxel clusters selected. This analysis comprised a separate three-way repeated measures ANOVA including task (Eriksen, Backmatching and Switching), task difficulty (easy vs. difficult) and hemisphere (left, right) for each of the ROIs. The multiple demand property was indicated by a region-specific main effect of task difficulty (Stiers et al. 2010).

### Voxel-wise task preference profiles

Co-activated voxels in the four regions of interest were the focus of all subsequent analyses. They were categorized according to their response strength during each of the three tasks into one of the several task preference profile categories. Unsmoothed data from data set 2, comprising the six runs of blocked task performances as described above, were used for this. Measures of response strength during task execution were derived from percent signal change maps computed from the estimated regressor weights in the first level event-related GLM analysis. In these six runs, there was no manipulation of task difficulty. For each task, a separate percent signal change image was computed over the six runs. These images were sampled to get for each of the selected voxels 3 signal change values, one for each task.

The task-specific percent signal change values were used to determine the task preference profile for each voxel. Six possible prototypical profiles were each represented by a binary vector in which each position represents one task and the binary value at each position indicates whether the corresponding task is being preferred or not by the voxel. Thus, [1 0 0] indicates preference for the Eriksen task, and [0 1 0] indicates preference for the Backmatching task, while [1 0 1] indicates preference for the Eriksen and the Response Scheme Switching task. For each voxel, the observed profile of task response strengths was correlated with each of the possible binary profile types. The type with the highest correlation was assigned to the voxel.

In parallel, a disjunctive *F*-contrast evaluating any pairwise difference between the three tasks, and computed from the single subject GLM analysis of the six runs (see above, “Six runs without task difficulty manipulation”), was used to establish whether the assigned task preference profile type was significant. This independent characterization of profile significance allowed us to conduct two analyses. The first analysis included only voxels for which the above *F* test was significant. While this focusses the analysis on voxels with a clear and meaningful difference in task responsiveness, it also reduced the number of voxels in the analysis. Therefore, a back-up second analysis was conducted which included all the multiple demand voxels regardless of whether the assigned profile was significant or not. This second analysis allowed us to observe whether this voxel reduction might have affected the analysis outcome.

Functional couplings between voxels are likely to become less specific if the voxels respond to more than one task, since the time courses of these voxels reflect activity in two or more neuron populations of different functional specialization. Therefore, our main interest was in voxels with a significant preference for one of the three tasks (i.e. a “mono-preference”). However, in parallel we also performed similar analysis including voxels of all six task preference types (multi-preference voxels).

This led us to conduct a total of four analyses organized along two dimensions: (1) mono-preference voxels or all preference type voxels, and (2) only voxels with significant preference profiles or all voxels regardless of the significance of task response differences. The largest number of voxels is obtained in the analysis including all preference types disregarding the significance of the profiles. While this analysis yields the highest statistical power, it also most likely comprises the functionally noisiest data set.

To demonstrate the reliability of the preference types, we performed a split half analysis in which the first three runs were used to assigned preference profile types as described above, while the last three runs were used to obtain an independent confirmation of the assigned types. The confirmatory analysis was performed for the voxels in each of the six preference type separately and consisted of 2nd level pair-wise comparisons on the percent signal change estimates for each of the three tasks. Voxels with a non-significant preference profile were excluded from the reliability analysis. For a particular preference type and for each participant, the average task-specific percent signal change was computed over the voxels of that type. These average values were normalized prior to entering the second level analysis to reduce the error variance. Repeated measures *t* tests were chosen and Bonferroni correction was applied within each analysis to reduce the chance of false positives.

### Voxel-wise functional coupling data

The commonly active voxels across four ROIs were selected because they showed a significant increase in BOLD signal contingent upon presentation of task trials in three different task paradigms. Despite their activation in all three tasks, the selected voxels differ in their response profile over these tasks. The question that we wanted to address is whether voxels with the same task preferences show a stronger functional coupling between them. The strength of functional coupling was quantified in different ways, which will be described below.

#### Task-related functional coupling

Trial-to-trial variation in response strength provides information on the strength of neural communication between the cell populations at the time of task execution. We refer to this trial-to-trial correlation in the BOLD response amplitude as the task-related functional coupling. To avoid circularity in our results, we eliminated the signal increase relative to baseline which is induced by the engagement during the task—this overall response strength measure was already used in the previous step to quantify task preference profiles. We epoched the unsmoothed time series data from all six runs from + 3 to + 7 volumes relative to the volume during which the first trial in a block started (i.e. from 4–6 s to 14–16 s). Then for each epoch, we subtracted from the value of the separate time points the average voxel signal over all five time points. This eliminated signal increase relative to baseline (i.e. the periods when no task was being performed). These standardized time courses from blocks of the same task were concatenated across runs, yielding per voxel and per task a time series vector showing the signal fluctuations during the task execution relative to zero. For each of the voxels in the selected ROIs, there were three such vectors, one for each task. For each of the three tasks, the task-related functional coupling vectors of all voxels were used to create a separate voxel-by-voxel Pearson correlation matrix. The correlation matrix was Fisher-*Z* transformed to correct for the non-normal distribution of the correlation statistic. From these matrices, voxel pairs could be selected based on their task preference profiles (for details, see subsection of the “Methods”, “Relating functional coupling of voxel pairs to their task preference”). This allowed us to investigate the dependency of coupling strength between voxels on their similarity or difference in task preferences.

#### Resting state functional coupling

In addition to task-related functional coupling, we quantified the strength of functional coupling outside of the time window of task execution, when the brain was at rest. For this, we used the third dataset of fMRI images acquired during 10 min of rest. These data were preprocessed as described above (see “Methods”, subsection “Handling of dataset 3: Resting state fMRI data”), retaining only the low-frequency (0.01–0.001 Hz) fluctuations in BOLD signal. For each of the selected commonly active voxels, a voxel-by-voxel low-frequency time course correlation matrix was computed using the Pearson correlation coefficient. The correlation matrix was than Fisher-*Z* transformed to correct for the non-normal distribution. From this matrix, subgroups of voxel pairs were selected in order to investigate a relationship between task preferences of the voxels and the strength of their low-frequency fluctuation coupling during a state of rest (for details, see subsection of the “Methods”, “Relating functional coupling of voxel pairs to their task preference”).

#### Resting state functional connectivity profiles

The resting state data were used in yet another way to study neural communication in relation to task preferences of individual voxels. For each of the selected commonly active voxels, a whole brain functional connectivity profile was computed. This connectivity profile is a vector of the Pearson correlation between the voxel’s low-frequency time course and similar time courses of all the grey matter voxels of the participant’s brain image. Next, for each of the selected commonly active voxels, a voxel-by-voxel functional connectivity profile similarity matrix was computed, with Eta^2^ as measure of similarity (Cohen et al. 2008). Subgroups of voxel pairs with same and different task preference profiles were selected from this matrix to investigate the dependency of whole brain functional connectivity similarity of commonly activated voxels upon specific task preferences (for details, see subsection of the “Methods”, “Relating functional coupling of voxel pairs to their task preference”).

### Relating functional coupling of voxel pairs to their task preferences

The subsequent investigation of task preference dependency of functional couplings between voxels was conducted in a similar matter for the different types of coupling data (task-specific functional coupling, resting state functional coupling, and resting state functional connectivity similarity). The analyses were performed on correlation matrices from individual participant data and are only integrated at the group level for the sake of ease of presentation. Correlation matrices comprising Pearson correlation coefficients were Fisher-*Z* transformed prior to entering the analyses to improve the parametric distribution of the values.

Voxel pairs were created by selecting one position on each dimension of a matrix and the value in the matrix cell defined by these two positions was the coupling or similarity value for that voxel pair. Subgroups of voxel pairs were created by selecting from the correlation matrix all the voxel pairs meeting particular requirements, such as the task preference type of the voxels in the pair, the significance of the differential task responsiveness, the ROI they were located in and for pairs located within the same ROI the Euclidean distance between the voxels in the pair. Because of the inherent local spatial smoothness of fMRI data, which exists even if the data are not explicitly spatially smoothed prior to analysis as in the current study, voxels at short distances from one another are more likely to exhibit more similar time courses than voxels further apart in space. This spatial relatedness does not pertain to the voxels located in different ROIs. Therefore, we conducted a separate analysis for voxel pairs in which the voxels were located in the same ROI and for voxel pairs where each element of a pair belonged to a different ROI. For the within ROI analysis, the voxel pairs were first ordered according to the Euclidean distance between the elements of each pair, and this ordered distance range was divided into 100 bins. Within each bin, voxel pairs from the class of same and different preference types were selected to ensure that the classes were matched for distance. This was started by selecting the class with the fewest pairs within the bin. For each pair in this least frequent class, a voxel pair from the other class was selected. This selection was biased towards a larger or equal distance for the same preference voxels, because only a smaller distance for this class would undermine conclusions regarding a stronger functional coupling between voxels in this class. If no such voxel was found, both pairs were excluded from the analysis. As a result, the same numbers of voxel pairs were selected within the two classes, matched for similar or larger Euclidean distance in the same preference class. Because this procedure considerably reduces the number of voxel pairs that can be entered into the analysis, a similar constraint was not imposed on the between ROIs voxel pairs, where the spatial signal contamination does not pose a problem.

### Voxel preference profile-specific whole brain functional connectivity

Starting from the single voxel whole brain functional connectivity profiles that were used to compute the connectivity profile similarity matrix (see subsection “Resting state functional connectivity profiles”, above), a statistical assessment was made of the task preference-specific functional connectivity profiles across participants. For each participant, each of the two IFS1 ROIs, the single voxel functional connectivity maps were average for voxels of the same mono-task preference type. This resulted in three FC maps per ROI and per participant. These average FC maps were Fisher-Z transformed and smoothed with a 6 mm FWHM Gaussian kernel to compensate for interindividual anatomical variation. These maps were used in a second level repeated measures GLM analysis with two ROIs (left and right) and three task preference types (Eriksen, Backmatching and Switching) as the independent factors. A general preference type-specific functional connectivity map was generated by a contrast that combined the left and right data into a single statistical image of voxels with an association strength that significantly differed from zero. The statistical image was corrected for multiple comparisons using a whole brain FDR-corrected significance level of 0.05.

## Results

### Behavioural data

The reaction time of the participants was significantly dependent both on the type of task [Eriksen, Backmatching, Switching: *F* (1.92, 21.01) = 8.1, *p* < 0.001] and on the difficulty level of the trials [Easy-data set 1, Difficult-data set 1, Difficult-data set 2: *F* (1.35, 14.89) = 44.6, *p* < 0.001]. However, there was a strong interaction between these two factors [*F* (2.26, 24.88) = 28.1, *p* < 0.010, Fig. 1e]. This was due to only a weak positive effect of difficulty in the Eriksen task [*F* (1.275, 14.026) = 10.9, *p* = 0.003] and in the Backmatching task [*F* (1.92, 21.11) = 0.8, *p* = 0.442], but a strong positive difficulty effect in the Switching task [*F* (1.91, 21.01) = 128.8, *p* < 0.001]. The easy Switching trials yielded on average the lowest reaction times, while the difficult Switching trials generated the largest reaction times of all conditions. At the level of data set 2, with only difficult task blocks, the reaction times for Switching (558.7 ± 82.1 ms) were significantly higher than those for Backmatching [497.2 ± 77.0; *t*(11) = − 3.9, *p* 2-tailed = 0.003] and for Eriksen [472.8 ± 60.6 ms; *t*(11) = − 6.1, *p* 2-tailed < 0.001]. The latter two did not differ significantly at the Bonferroni corrected significance level [*t*(11) = − 2.0, *p* 2-tailed = 0.069].

A somewhat comparable pattern of results was observed when analysing the percentage of errors made, although in this case the interaction between task type and difficulty level only reached borderline significance [*F*(4, 44) = 6.5, *p* = 0.062]. Nevertheless, post hoc analyses learned that there was no significant effect of difficulty on the percentage of errors made in the Eriksen task [*F* (2, 22) < 0.1, *p* = 0.991] and the Backmatching task [*F* (2, 22) = 0.1, *p* = 0.932], but a strong positive effect was observed during the Switching task [*F* (2, 22) = 10.4, *p* = 0.001, Fig. 1f]. For data set 2, with only difficult task blocks, the percentage of errors was significantly lower in the Eriksen task compared to both Backmatching [*t*(11) = − 3.4, *p* 2-tailed = 0.006] and Switching [*t*(11) = − 3.9, *p* 2-tailed = 0.002], which did not differ [*t*(11) = 0.4, *p* 2-tailed = 0.685].

In summary, while the manipulated difficulty level had no or only a small effect on the reaction times and the number of errors made for the Eriksen task and for the Backmatching task, it clearly affected performance on the Response Scheme Switching task, with increased reaction times and more errors in the difficult blocks. Therefore, only for the Switching task there was clear behavioural evidence that our manipulation increased the difficulty. When taking the perspective of inter-task differences in difficulty, the reaction times in data set 2 showed that Backmatching and Eriksen required equal response times, whereas Response Scheme Switching required significantly larger response times, probably reflecting increased motor conflict and selection (Wong et al. 2015). In contrast, the percentage of errors made suggests that Eriksen was the easier task, while Backmatching and Switching were equally more difficult.

### Region of interest delineation

**Fig. 2:**
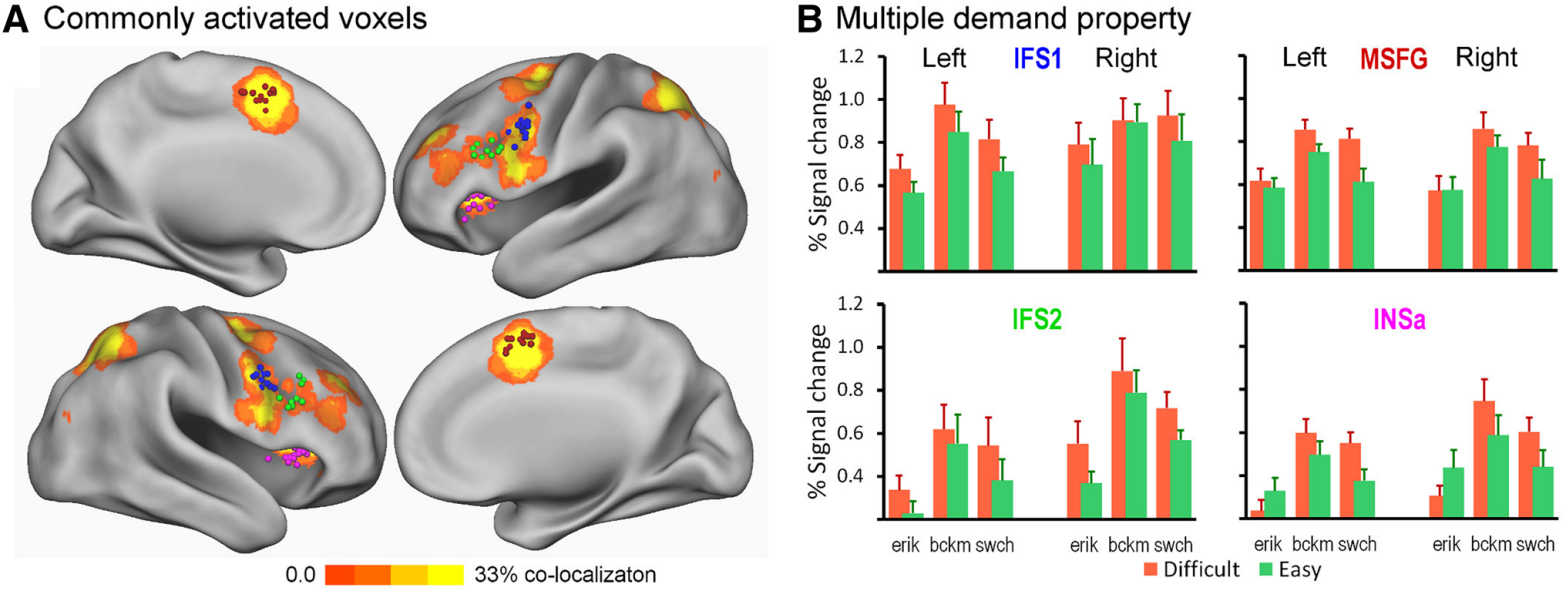
Identification of commonly activated multiple demand regions. **a** Regions of the brain where voxels are commonly activated by the three cognitive tasks. The graded colour map shows the amount of overlap across participants in the first level conjunction maps (*F*-statistic) of activation in the three tasks. The colour range is from zero (no colour = no significant voxel) to yellow (significant in 1/3rd of participants). The spherical foci mark the local maxima of the conjunctive activation in individual maps that were chosen as the center to delineate the individual regions of interest (ROI). Thus, each focus is the center of the ROI of a particular participant. The four classes of individual ROIs are distinguished by four different colors; IFS1 = blue, IFS2 = green; MSFG = red; INSa = fuchsia. **b** Multiple demand property of the four classes of ROIs. While part **a** shows more than four regions that are commonly activated by the three tasks, not all the commonly activated regions have the multiple demand property—i.e. showing increased activation with increased task difficulty regardless of task content. Each graph presents the percent signal change, averaged across participants (error bars indicate + 1 SE), in each task and each level of task difficulty, with green bars representing activity during easy trials and orange bars during difficult trials. In ISF1, IFS2 and MSFG, the percent signal change is systematically higher in difficult conditions compared to easy conditions, resulting in a significant main effect of difficulty level, not interacting with the type of task (see text for details). In INSa, the activation pattern is reversed for the Eriksen flanker task, yielding a significant interaction between task and difficulty level, with no main effect of task difficulty

Voxels that were commonly activated by the three tasks were identified using task data set 1. In each of the 12 participants, clusters of voxels that were significantly activated by all three tasks (pooling over the easy and difficult conditions) were found in the vicinity of the a priori selected anatomical locations of interest at least in one, but mostly in both hemispheres. These voxel clusters observed in individual data sets, summarized in Table 1 and visualized in Fig. 2a, are the regions of interest for the current study.

To confirm that these commonly activated voxel clusters exhibited the multiple demand property, we compared their percent signal change during execution of easy and difficult trial blocks in each task. It was found that regardless of the task, anatomical region or hemisphere, difficult trials induced higher signal change in these voxels compared to easy trials [*F* (1,11) = 26.8, *p* < 0.0001]. Modulation of the BOLD signal by task difficulty was also examined for each anatomical location separately (see Fig. 2b). For three of the four locations, there was a significant main effect of task difficulty. For the INSa ROIs, however, the main effect of task difficulty did not reach significance [*F*(1, 9) = 2.67, *p* = 0.1367]. This was due to a reversed difficulty effect in the Eriksen task compared to the other two tasks (Fig. 2b, lower right panel), which lead to a significant interaction between task and difficulty level [*F*(2, 22) = 7.2, *p* = 0.005]. This interaction term was not significant for the other ROIs or for the global analysis over anatomical locations. For the INSa voxels, the difficulty modulation in the Backmatching and the Switching tasks was significant [smallest *t*(11) = 2.75, *p* = 0.0189].

This analysis allows us to conclude that the four regions, in addition to common activation by the three tasks, also show a multiple demand activity, although in the anterior insula the functional characterization appeared more complex than in the other three regions. This consistent finding is despite the less consistent effect of these difficulty manipulations on the behavioural measures of response time and errors made (see “Results” section, subsection “Behavioural data”).

### Task preferences in commonly activated voxels

While all the selected voxels were active during the three tasks, this does not imply that they were equally activated by the three tasks. The task-specific response strength of the selected voxels was quantified using the six runs of data set 2, with only trials of the difficult type. Because we wanted to look at individual voxel responses no smoothing was applied prior to analysis. The percent BOLD signal change induced by trials of each task was estimated per voxel from first level GLM analyses that combined all the six runs of one participant. This yielded for each participant per voxel three percent signal change values, one for each task.

Voxels were categorized in discrete task preference types based on their percent signal change profile over the three tasks. First, a global any-difference *F* test was used to establish for each voxel whether its response strength was significantly modulated by the type of task being performed. If this test was not significant for a particular voxel, that voxel was considered to have no task preference. Second, the voxels that did show a significant task modulated response were categorized into discrete classes depending on the shape of their task response profile. Thus, a voxel with a higher percent signal change response during the Eriksen task was placed in the category [1 0 0], while a voxel preferring switching was categorized as [0 0 1], and a voxel preferring both was placed in [1 0 1], etc. The categories were assigned by correlating a voxel’s observed percent signal change profile with each of these prototype vectors (six in total), and assigning the voxel to the category with the highest prototype vector correlation (see “Methods”, subsection “Voxel-wise task preference profiles”).

**Fig. 3:**
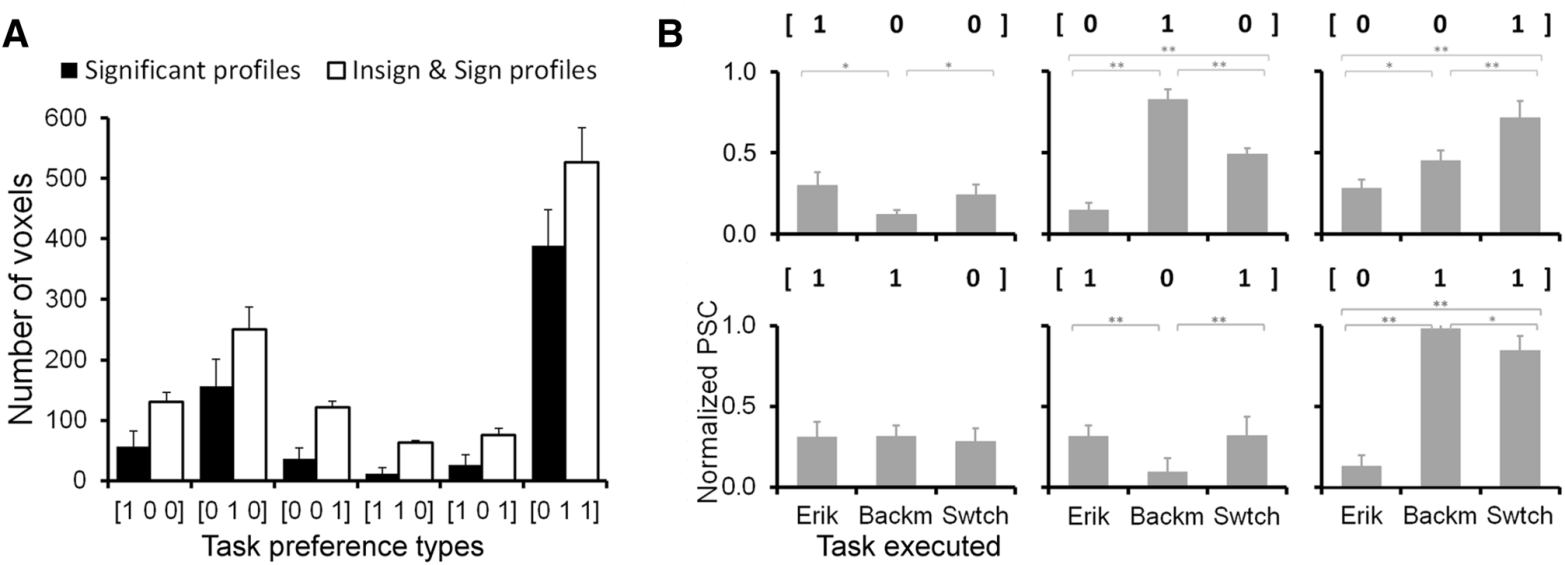
Frequency distribution and reliability of task preference profiles. **a** Numbers of selected voxels, averaged over participants, assigned to each of the task preference profiles, when only voxels are considered with a significantly different response strength between tasks (black bars) and when all voxels are considered regardless of whether task differences were significant (white bars). Error bars indicate + 1 SE. **b** Normalized percent signal change induced by each of the three tasks in voxels assigned to different task preference profiles. Only voxels with significant task profiles were included. The assignment to preference types was based on data from the first three runs of the task, while the percent signal change was estimated from the last three runs of the same task. Error bars indicate + 1 SE. **Significant at the Bonferroni corrected significance level, *significant without correction for multiple comparisons

The distribution of voxels over the six possible task preference types is summarized in Fig. 3a. Averaged over participants, the mean number of selected voxels across all four ROIs was 1168.3 (± 297.3), with a minimum of 821 and a maximum of 1599. The average number of voxels with a significant preference profile was 675.0 (± 242.5), or 42.2 ± 14.1% of selected voxels, with a minimum of 19.4% and a maximum of 69.3%. This means that on average 493.3 (± 210.5) voxels did not show a significant preference for one or other of the three tasks. As can be seen in Fig. 3a, within the mono-preference types (e.g. [1 0 0], [0 1 0], etc.) the voxels with a stronger Backmatching response were most frequent. For the dual preference types, the voxels preferring both Backmatching and Switching were the most frequent. The distribution patterns were very similar when considering all voxels, or only the voxels with a significant task preference profile. The distribution of voxels within each ROI separately over the different preference types is described in Table S1.

To demonstrate the reliability of the task preference assignment, we applied a split-half validation (Baker et al. 2007), in which we used the first three runs of dataset 2 to assign voxels to task preference profiles, and the last three runs to independently study the percent signal change for the three tasks. Only voxels with a significant task profile were included. This analysis shows that the profiles assigned to the voxels based on data from the first three runs are on average well replicated in the percent signal change data (Fig. 3b). The results confirm the expectations for four of the six profiles. For voxels of the [1 0 0] type, the percent signal change data only partially confirm the preference type, while for voxels of type [1 1 0], which are the least frequent category (cf. Fig. 3a), the profile is not confirmed.

### Task-related functional coupling

**Fig. 4:**
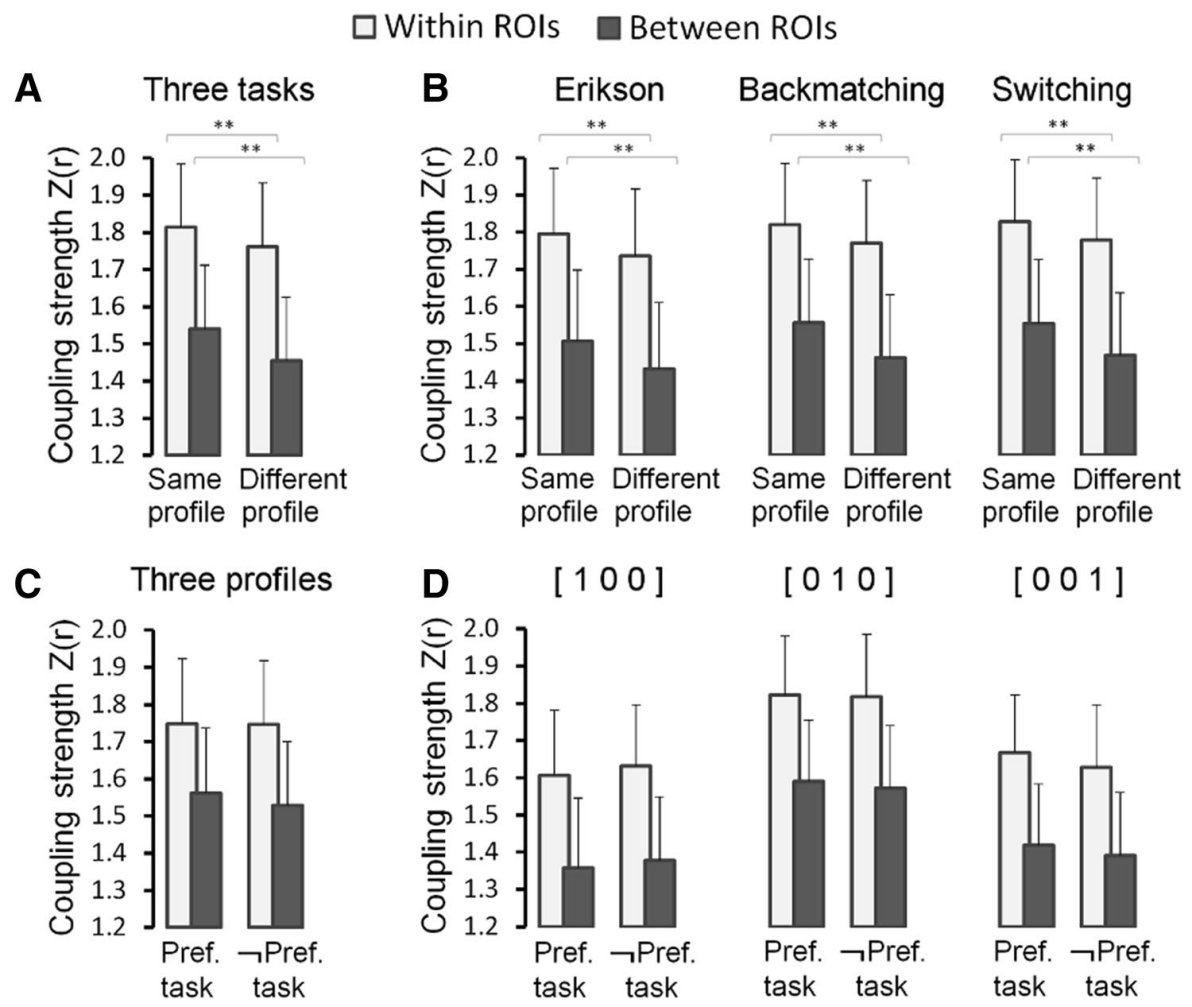
Task-based (high-frequency) functional coupling (Fisher-*Z* transformed Pearson correlation) between multiple demand voxel pairs with significant task profiles preferring one task over the other two (mono-preference). **a** Functional coupling between pairs of voxels preferring the same task (same preference profile) and pairs of voxels preferring different tasks (different preference profile); data are pooled across the three tasks. **b** Same data as in **a** presented for each task separately. **c** Functional coupling between voxels with the same task preference, when the task being executed is the preferred task (“Pref. task”) or when the task being executed is not the preferred task (“¬Pref. task”), pooled across preference profiles. **d** Same data as in **c** presented separately for voxel pairs preferring the Eriksen task (“[1 0 0]”), the Backmatching task (“[0 1 0]”), and the Switching task (“[0 0 1]”). Error bars indicate + 1 SE. **Significant at the Bonferroni corrected significance level, *significant without correction for multiple comparisons

The basic idea investigated here is that multiple demand voxels that are more active during one or more tasks compared to others, and hence share a task preference, reflect interconnected neuron populations within and across network nodes that are recruited by specific demands of these tasks. We want to show that these interconnections exist, and can be traced in functional connectivity data, independent of the execution of the tasks. Before doing so, however, it needs to be confirmed that these connections exist during the execution of the tasks. Therefore, we quantified the trial-to-trial variation in BOLD amplitude strength during execution of the three tasks and computed how well these correlated between the selected multiple demand voxels. The results show that the strength of these functional couplings depends on the task preferences of the voxels. The results for voxels with a significant preference profile favouring one task over the other two (mono-preference voxels) are summarized in Fig. 4a. Voxels preferring the same task exhibit a significantly stronger coupling between their response amplitude fluctuations than voxel pairs preferring a different task. This was the case for pairs in which each voxel was located in a different one of the eight ROIs and for pairs of voxels located in the same ROI. In the latter case, the pairs were chosen in such a way that the Euclidean distance between the voxels in the pairs was equal or larger in same preference voxel pairs compared to different preference voxel pairs [same preference: *M* = 7.92 ± 1.20 mm; different preference: *M* = 7.91 ± 1.19 mm; *t*(11) = 4.4, 1-tailed *p* = 0.9995]. The stronger functional coupling in same preference profile voxel pairs was also evident when data from each task were analysed separately (Fig. 4b). Moreover, the results were also similar when different criteria for voxel pair selection were used, such as when in addition to mono-preference voxels (e.g. [1 0 0], etc.) also voxels were considered that preferred two tasks (e.g. [1 1 0], etc.), or when also voxel were included in which the preference profile was not significant (see lower panel of Table S2).

We also looked at the strength of task-induced functional coupling between same preference voxels, when they were engaged by their preferred task and during execution of the tasks that they did not prefer. This analysis revealed that the coupling did not become weaker when the participant engaged in a task that was not the voxels’ preferred task. The results are summarized for the significant mono-preference voxels in Fig. 4c–d. Even without a correction for multiple comparisons, we did not find a significant difference in the coupling strength computed from the trials of the preferred task, compared to trials from the non-preferred task, neither in the voxel pairs spanning different ROIs nor in the voxels located in the same ROI. This result was also not dependent on the inclusion or not of only mono-preference voxels, or voxels of all preference types, or the inclusion of voxels with statistically non-significant task preference profiles (see Table S3). The stability of the functional coupling between same task preferring voxels regardless of the task being performed is a first indication that their coupling is not merely a temporal configuration, but the result of anatomical connections between the neuron populations responsible for the task preference observed in these voxels. Our functional coupling analyses during rest corroborate this suggestion.

### Functional coupling during rest

**Fig. 5:**
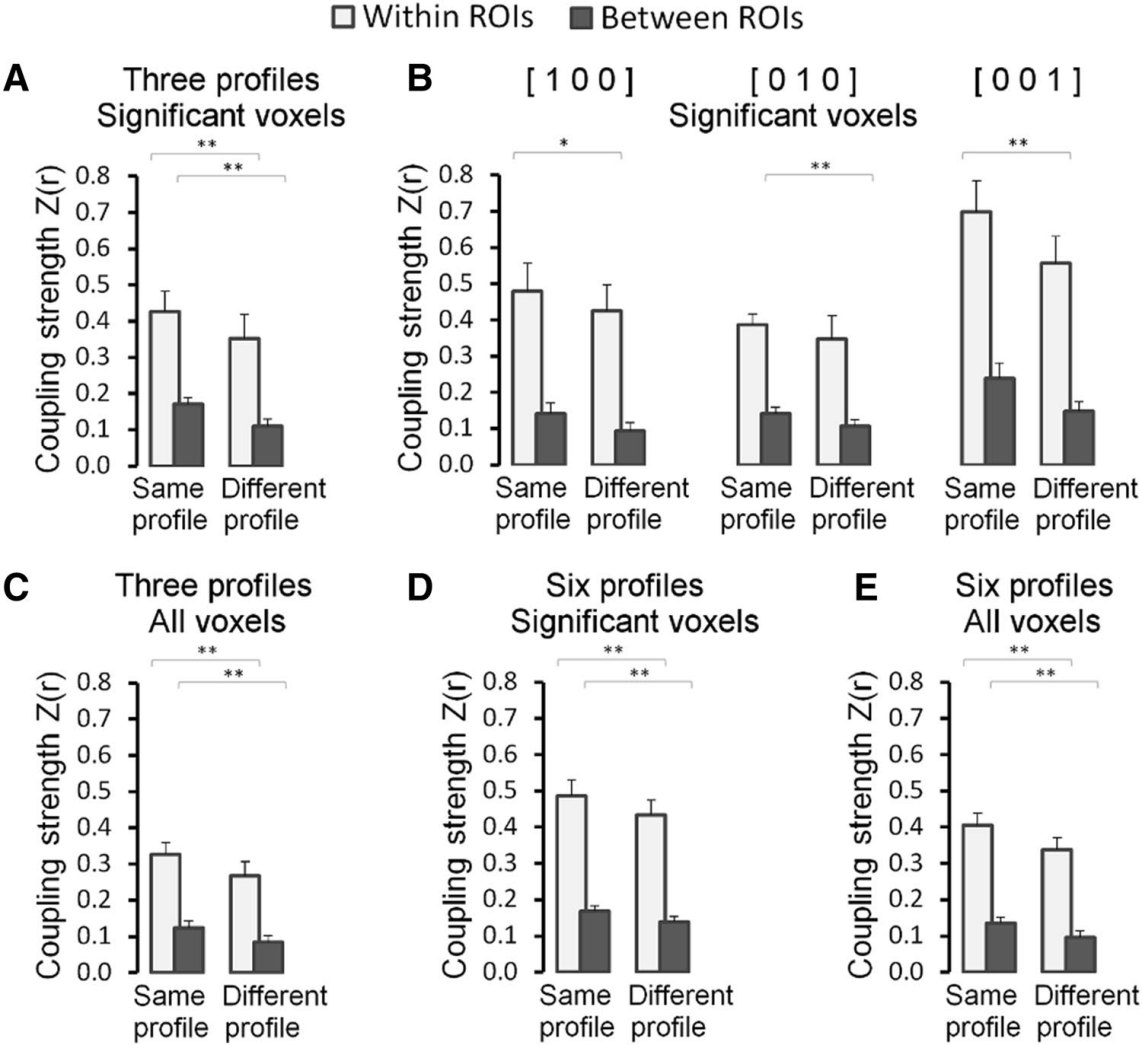
Low-frequency (resting state) functional coupling (Fisher-*Z* transformed Pearson correlation) between multiple demand voxels in relation to task preference similarity. **a** Functional coupling between pairs of voxels significantly preferring one task over the other two (mono-preference). Pooled over the three task preference profiles, pairs of voxels with the same preference profile (“same profile”) show significantly stronger functional coupling during rest than pairs of voxels preferring different tasks (“different profile”). **b** Same data as in **a** presented for each preference type separately: voxels preferring the Eriksen task (“[1 0 0]”), the Backmatching task (“[0 1 0]”), and the Switching task (“[0 0 1]”). **c** Similar data as in **a**, but including all voxels preferring one task over the other two (mono-preference) regardless of whether the profile was statistically significant. This lenient inclusion criterion increases the number of voxels in the analyses and statistical power. **d** Similar data as in **a**, but including additionally voxels with significant profiles that preferred two tasks over the third task. **e** Similar data as in **a**, but including all six task preference profiles regardless of the significance of the profile. Error bars indicate + 1 SE. **Significant at the Bonferroni corrected significance level, *significant without correction for multiple comparisons

The stability over time of functional couplings between multiple demand voxels was examined in resting-state fMRI data collected in an independent run preceding the acquisition of dataset 2. Low-frequency signal couplings in resting state data are thought to reflect structural connections between neuronal populations established by repeated use (e.g. Margulies et al. 2009; Miranda-Dominguez et al. 2014). Therefore, if the functional coupling patterns observed between voxels during task execution reflect established anatomical connections, these patterns should be observed also in the low-frequency couplings of their activity during rest. The resting-state functional connectivity analyses are summarized in Fig. 5 and detailed in Table S4. Pooling together the data from the voxels with a significant task preference profile preferring one task over the other two (mono-preference profiles), voxels with the same task preference show a significantly stronger low-frequency functional coupling than similar voxel pairs with different task preferences (Fig. 5a). This was the case for pairs of voxels located in the same ROI [*t*(11) = 3.6, *p* < 0.01] matched for inter-voxel Euclidean distance, and for voxels located in different ROIs [*t*(11) = 7.6, *p* < 0.01]. The results for the three different types of mono-preferences are in the same direction (Fig. 5b), but somewhat less strongly, with some effects not reaching statistical significance. However, the strong effect in the pooled analysis was replicated regardless of whether all preference types were included or whether the voxels with non-significant preference profiles were included or not (Fig. 5c–e).

**Fig. 6:**
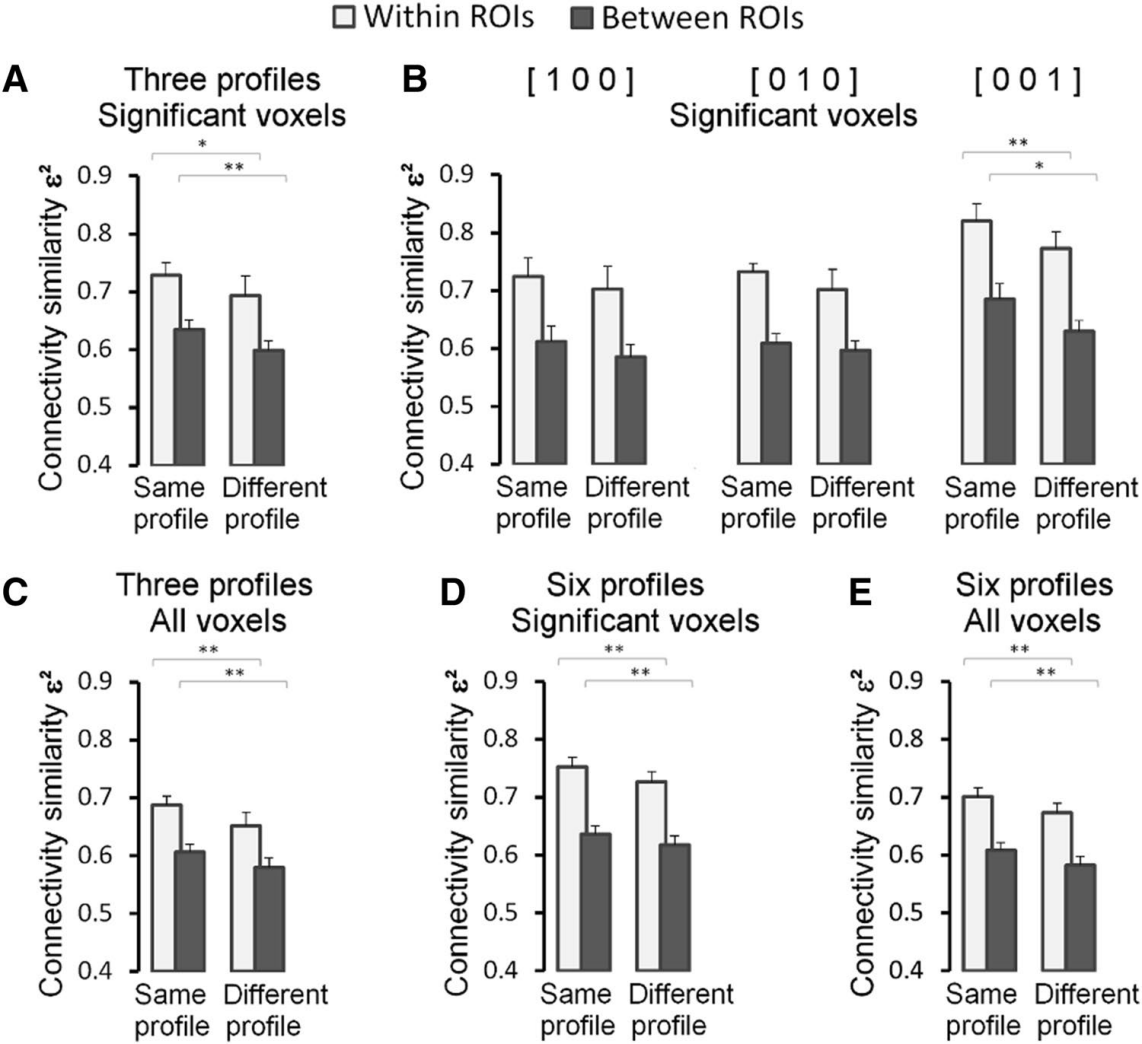
Similarity (eta^2^) in whole brain functional connectivity profiles (low-frequency fluctuations in resting state signal) between multiple demand voxels as a function of similarity in their task preferences. **a** Multiple demand voxels with significant task profiles preferring one task over the other two (mono-preference). Pooled over the three task preference profiles, pairs of voxels preferring the same task (“same profile”) have significantly higher while brain connectivity similarity than pairs of voxels preferring different tasks (“different profile”). **b** Same data as in **a** presented for each preference type separately: voxels preferring the Eriksen task (“[1 0 0]”), the Backmatching task (“[0 1 0]”), and the Switching task (“[0 0 1]”). **c** Similar data as in **a**, but including all voxels preferring one task over the other two (mono-preference) regardless of whether the profile was statistically significant. This lenient inclusion criterion increases the number of voxels in the analyses and statistical power. **d** Similar data as in **a**, but including additionally voxels with significant profiles that preferred two tasks over the third task. **e** Similar data as in **a**, but including all six task preference profiles regardless of the significance of the profile. Error bars indicate + 1 SE. **Significant at the Bonferroni corrected significance level, *significant without correction for multiple comparisons

A plausible reason for this configuration of segregated subnetworks of interconnected neuron populations is that their functional specialisation requires that they receive input from different source regions and project to different target regions. Different tasks involve different types of sensory input, require attention to different aspects of this input and demand specific processing of it. Hence, depending on the task executed, different remote regions target different subpopulations of neurons within the multiple demand regions. To investigate this hypothesis, we used the resting state functional connectivity data to compute a whole brain functional connectivity profile for each multiple demand voxel. Next, whole brain connectivity similarity was quantified by computing the voxel-to-voxel correlations between their functional connectivity profiles. We predicted that voxels with similar task preferences had more similar whole brain functional connectivity profiles than voxels with different task preferences. The results of this analysis for the mono-preference voxels with significant profiles are summarized in Fig. 6a (see details in Table S5). As predicted, the similarity in functional connectivity was significantly higher for voxels preferring the same task compared to voxels preferring a different task. This is true for voxels located in the same network node (with matched inter-voxel distance) and for voxels located in different nodes of the network. While the effect was in the same direction for each of the preference profiles separately, statistical significance was reached only for the voxels preferring the Switching task (Fig. 6b; see statistical details in Table S5). When the voxels were pooled across preference types, however, the effect was convincingly strong regardless of whether all preference profiles were included or whether the significance of these profiles was taken into account (Fig. 6c–e).

### Visualization of task preference-specific connectivity profiles

**Fig. 7:**
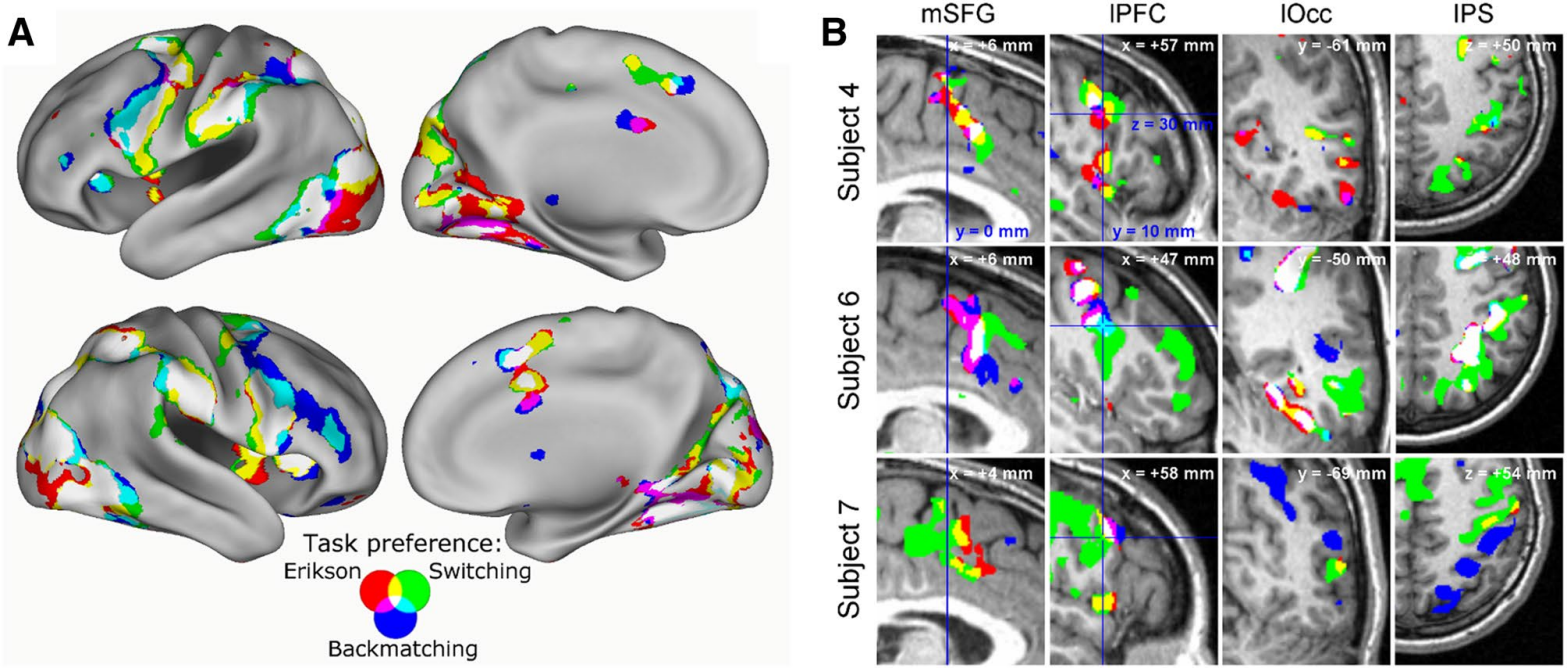
Functional connectivity profiles of multiple demand voxels in IFJ. **a** Group summary. Statistical maps of functional connectivity of voxels in left and right IFJ that share the same task preference, either for the Eriksen task (red), the Backmatching task (blue), or the Response Scheme Switching task (green). Each map was thresholded at a *t* value of 3.25, corresponding to an uncorrected single voxel significance level of 0.001. However, visualized data were significant at the FDR-corrected significance level of 0.05, which is a correction for multiple comparisons at the whole brain level. While there are several areas where all the IFJ voxels show significant functional couplings (colored white), there is also clear differentiation between the three task-preference type with respect to the cortex to which they show a significant functionally connection. These patterns of only partially overlapping connectivity profiles reflect what is consistent across the 12 participants. **b** Details of individual functional connectivity in three representative participants. Shown are average connectivity strength maps [*Z*(*r*) > 0.3] of voxels in right IFJ preferring the Eriksen, the Backmatching, or the Switching task (same colour scheme as in **a**). *mSFG* medial superior frontal gyrus, *lPFC* lateral prefrontal cortex, *lOcc* lateral occipital cortex, *IPS* intraparietal sulcus

To further validate the hypothesis that multiple demand voxels with a particular task preference represent a unique pattern of functional connections to the multiple demand area, we asked whether this unique connectivity profile could be identified across participants. To answer this question, a voxel-wise GLM analysis was conducted with the average mono-preference functional connectivity maps, computed for each participant separately, as dependent variable. Since it is not likely that voxels in different multiple demand areas have the same profile of connections, we focused this analysis on the mono-preference voxels in inferior frontal junction (IFJ), the largest voxel cluster investigated. Figure 7a shows the cortical distribution of the grey matter voxels that are significantly functionally coupled with left and right IFJ voxels across the 12 participants, as a function of the task preference profile of the IFJ voxels. While a core of brain regions are commonly functionally associated with each of the three classes of multiple demand voxels, there are also substantial areas where only two or only one of the three subclasses of voxels show significant functional coupling.

The overall lay-out of regions functionally coupled with the IFJ voxels reflects the spatial lay-out of the task-positive network (Dosenbach et al. 2007; Fox et al. 2005). First, in the peri-IFJ area the coupled voxels covered the precentral gyrus and sulcus (premoter cortex) and extending into the central sulcus’ (primary motor cortex) hand representation. For IFJ voxels preferring Switching or Eriksen, the regions of significant coupling extended more posteriorly, and more ventrally to BA 44 and into the sensorimotor regions of mid-insula. For IFJ voxels preferring Switching and Backmatching, the coupling extended to the anterior bank of the precentral sulcus and further into the inferior frontal sulcus (particularly left). Second, functionally connectivity regions included two of the other multiple demand nodes studied here: anterior insula and preSMA. The last node, in anterior inferior frontal sulcus, was only coupled to IFJ voxels preferring Backmatching and Switching, but not Eriksen, which may reflect differences in task complexity (Badre and D’Esposito 2007; Crittenden and Duncan 2013). In contrast, the dorsolateral PFC and specifically region BA46, which are also considered MD (Woolgar et al. 2011), showed coupling with IFJ voxels preferring Eriksen, but only very few of those preferring the other two tasks. Thirdly, the IFJ coupled areas also incorporated the frontal-parietal-collicular attentional priority maps network (Womelsdorf, and Everling 2015) (bilaterally FEF, IPS and superior colliculi), with the coupled region for all three voxel classes extending through the IPS to the dorsal visual system including the hMT + area. Fourth, the coupled region further included retinotopic visual areas in the dorsal and ventral occipital lobe, as well as lateral and ventral occipital–temporal cortex associated with visual form analysis. Remarkably, Eriksen preferring IFJ voxels were coupled with a larger area of occipital visual cortex than the other two tasks, possibly reflecting the need in this task to supress interference from the surrounding distractors. Fifth, there was a strong coupling of IFJ voxels to the anterior supramarginal cortex. Although it is co-activated across cognitive tasks, this region is directly linked to the ventral premotor cortex (Kelly et al. 2010) and exhibits motor action sensitivity without the multiple demand property (Stiers et al. 2010). In addition, the IFJ voxels were also functionally coupled with voxels in the orbital frontal cortex (in and lateral to the medial orbital sulcus) with only minimal overlap across the three voxel classes. And lastly, the IFJ voxels showed significant functional coupling with subcortical structures (not shown in Fig. 7). All three IFJ voxel classes showed only partially overlapping coupling with the putamen (MNI ± 22, 2, 7), extending anteriorly to caudate/putamen (± 14, 8, 7). Moreover, all three voxel types were coupled with the posterior–ventral thalamus (± 8, − 21, − 1), the lateral geniculate nucleus (± 16, − 28, 1) and the superior colliculi (± 4, − 27, − 4).

While group level functional connectivity data emphasize central tendencies in brain organisation that are shared across individual brains, they obscure the more detailed organisational features of networks (Braga and Buckner 2017). Therefore, Fig. 7b shows a selection of detail views on the voxel task preference-related differential connectivity patterns in individual data from three representative participants. These details reveal a more variable and patch-like organisation of the connectivity pattern compared to the group level statistical images, associated with different subclasses of IFJ voxels preferring a specific task. This pattern of variation in the strength of the functional coupling of the three subclasses of IFJ voxels suggests a variation across multiple demand voxel classes in the regions with which they exchange information. A subcomponent of this variation is consistent across participants.

## Discussion

We investigated the stability in functional organization of four multiple demand regions in the prefrontal cortex. We confirmed the earlier finding that although the voxels in these regions significantly activate during different tasks, individual voxels reliably show stronger responses during execution of particular tasks than other tasks. As shown in Stiers et al. (2010), the voxels with specific task preferences are distributed throughout the multiple demand regions. The main analyses were focused on the functional couplings between voxels in relation to their specific task preferences. To disentangle spatial separation of voxels from their functional coupling, we either controlled the distance between voxels within one node, or looked at voxels located in different nodes.

The analyses revealed that during task execution voxels with the same task preference have stronger functional couplings. This is regardless of whether the task executed is the preferred task or not. These couplings express the trial-to trial amplitude variations around the mean task-induced BOLD response. Thus, while these voxels overall respond less strong during execution of none preferred tasks, the trial to trial co-fluctuation of their activity level remains equally strong regardless of the task that is executed. This particular aspect of their functional coupling suggests that the coupling reflects not just a temporary exchange of information, but that it is also based on more stable connectivity patterns. While neurophysiological recordings show that over an array of monitored neurons a particular activity pattern disappears when the task phase is over (Lapish et al. 2008; Sigala et al. 2008; Stokes et al. 2013), these recordings centre on the information load carried by the activity of the neurons: does the population response allow to predict a particular task context? This activity content ceases to exist when the neurons in the assembly are no longer active—and their activity no longer increases the BOLD signal. But the particular wirings that allowed them to act as an assembly remain and are reflected in the co-fluctuation of their idle state activity over time.

In accordance with this interpretation, our further analyses revealed that the observed functional coupling patters are also present in the low-frequency fluctuations of the BOLD signal at rest, i.e. when participants are no longer engaged in the cognitive tasks. Even more than the coupling data during task execution, this result suggests that the voxels that are more engaged during execution of a particular task form stable subnetworks throughout the multiple demand network independent of task execution. These subnetworks are task specific and seem to be specialized for the particular demands and requirements of a concrete task. Our result confirms the earlier report by Waskom and Wagner (2017) that lateral prefrontal cortex voxels coding for particular task relevant stimulus features show stronger functional connectivity during subsequent rest. We here show that this is also true for the subsets of voxels from the multiple demand networks, both within and between the nodes of this network.

Lastly, we found that the voxels forming these subnetworks have significantly more similar whole brain functional connectivity profiles across participants. This finding suggests that subnetworks within the multiple demand network reflect different patterns of inflow and outflow of information—comparable to how in sensory areas spatially different subregions are defined by the different origin of their sensory input (e.g. different loci in retina or parts of the body). Different tasks involve different types of sensory input, require attention to different aspects of this input and demand specific processing of it. Hence, depending on the task being performed information received by the multiple demand regions is likely generated in different peripheral source regions and sent to other target regions. The axons coming from the remote cortical regions likely target different subpopulations of neurons within these multiple demand regions, which may be also spatially segregated, since invasive tract-tracing studies have revealed a “stripe-like” organization of projection neurons in spatially interdigitated patterns in the areas of the association cortex (Pucak et al. 1996; Marconi et al. 2001; Selemon and Goldman-Rakic 1988). It seems, therefore, that not only a cortical field is defined by its incoming and outgoing connections (Krubitzer 1995; Passingham et al. 2002), but that the same holds for subpopulations of neurons within a field (Selemon and Goldman-Rakic 1988; Park et al. 2017). As such, our results further substantiate the notion of connectional heterogeneity at the subregional level. Multivariate functional connectivity studies have delineated several large-scale brain networks that break up the cerebral cortex in vast functional territories (Beckmann et al. 2005; Damoiseaux et al. 2006; Kiviniemi et al. 2009). In vivo parcellation studies have shown that connectivity similarity analyses allow to further break up these territories into smaller fields, each with a unique connectivity profile resembling the organization of the larger network to which they belong, but uniquely different across individuals and species (Goulas et al. 2012, 2017; Sallet et al. 2013; Braga and Buckner 2017). Our current findings, together with those of some other studies (Park et al. 2017; Waskom and Wagner 2017), go a step further by showing that even within this connectionally more homogeneous fields, unique and function-specific subpatterns of connectivity can be demonstrated to exist.

As discussed above, it has repeatedly been demonstrated that the neural assemblies formed to encode task contexts, with their specific stimulus categories, response alternatives and mapping rules, are dynamic in nature. This means that they can no longer be demonstrated to exist in the functional responses within an assembly outside of the particular task phase. The most temporally restricted interpretation of this dynamic feature of assemblies is that they exist only for the short time that they can be traced. They are expressed in a fixed array of neurons that can randomly represent any task context for the time that it is needed, without leaving any trace of it afterward. Our results do not support this interpretation. Our study shows that the assemblies remain traceable in the functional coupling characteristics during execution of another than the preferred task and even in low-frequency BOLD fluctuations during rest. This raises the question how long they are traceable. While there is ample evidence that low-frequency functional coupling is constrained by anatomical connections (Johnston et al. (2008); Margulies et al., 2009; Miranda-Dominguez et al. 2014), several studies observed local task performance induced changes in functional connectivity of the areas engaged during the performance (Gordon et al. 2014; Hasson et al. 2009). Such results raise the possibility that the functional couplings during rest observed here are only temporal alterations in coupling that merely reflect the network dynamics of the task performed just earlier. A first point against this interpretation is that in our paradigm all three tasks were administered intermingled in short blocks. Therefore, each of the observed task-specific coupling patterns at least survived the execution of and switching between the other two tasks. A second observation supporting a larger temporal stability of the coupling patterns is that in nine of our 12 participants the resting state data were collected at the beginning of the second scanning session, which was after an interval of 5–15 days in eight of them. This suggests that the task-related patterns were stable over a considerable time window, and not just temporal changes induced by the just preceding tasks. Such longer lasting changes of functional connectivity have been demonstrated in the context of visual learning (Urner et al. 2013).

An even more intriguing question is whether the observed coupling patterns are induced by these specific three tasks, or whether they reflect stable processing paths established over longer time spans of engaging into similar types of cognitive activity. This would provide an interesting perspective on the implementation of cognitive functions or specific cognitive demands in the multiple demand network. It would imply that specific cognitive demands recruit dedicated subpopulations of neurons—assemblies that exist in parallel and constitute unique subnetworks that span the entire multiple demand network. This interpretation of the observed functional connectivity features would require that these couplings were already present before the participants learned to perform the three specific study tasks. While in most of our participants the resting state data were collected after learning to perform the tasks, in two of our participants these data had already been collected prior to entering the study. In both of them, the voxels, selected after they had been trained and had practiced for some time these three tasks, showed the task-specific functional coupling patterns already before the participant started to train on the tasks (see supplementary information, Table S6). While this is only a fragmentary observation, it does point out the direction for intriguing and promising future studies.

## Electronic supplementary material

Below is the link to the electronic supplementary material.

Supplementary material 1 (DOCX 61 KB)

## Footnotes

1 It should be noted that these two views do not need to be mutually exclusive, and that coding equipotentiality may exist in some neuron arrays, whereas task-specific changes in interconnections are formed in other neuron populations, located in different areas or maybe even in subpopulations within one area.
