# Supplementary material for: Functional connectivity of task context representations in prefrontal nodes of the multiple demand network
Source: Brain Struct Funct. 2018 Mar 3;223(5):2455–73. doi: 10.1007/s00429-018-1638-9 (PMC5968070; doi:10.1007/s00429-018-1638-9)
Supplement: Supplementary file 1 — Supplementary material 1 (DOCX 61 KB) [file 429_2018_1638_MOESM1_ESM.docx]

# Supplemental information

Table S1: Distribution of voxels in multiple demand areas over the different task preference profile types, for each ROI separately and pooled over ROIs, and when considering voxels without a significant task preference profile as a separate preference type (i.e., no preference) (see upper panel), or merging them with the significant voxels (lower panel).

|  |  |  |  |  |  |  |  |  |  |  |  |  |  |  |  |  |  |  |  |  |  |  |  |  |  |  |  |
| --- | --- | --- | --- | --- | --- | --- | --- | --- | --- | --- | --- | --- | --- | --- | --- | --- | --- | --- | --- | --- | --- | --- | --- | --- | --- | --- | --- |
|  |  |  |  |  |  |  |  |  |  |  |  |  |  |  |  |  |  |  |  |  |  |  |  |  |  |  |  |
| Preference |  | IFS1 left | |  | IFS1 right | |  | IFS2 left | |  | IFS2 right | |  | INSa left | |  | INSa right | |  | MSFG left | |  | MSFG right | |  | All ROIs | |
| Type |  | M | SD |  | M | SD |  | M | SD |  | M | SD |  | M | SD |  | M | SD |  | M | SD |  | M | SD |  | M | SD |
| *Separate type for non-significant profiles* | | | | | | |  |  |  |  |  |  |  |  |  |  |  |  |  |  |  |  |  |  |  |  |  |
| [1 0 0] |  | 22.5 | 19.3 |  | 14.8 | 14.7 |  | 5.0 | 10.6 |  | 2.3 | 3.7 |  | 0.0 | 0.0 |  | 0.1 | 0.3 |  | 9.4 | 24.6 |  | 2.4 | 6.6 |  | 56.5 | 55.6 |
| [0 1 0] |  | 9.8 | 11.2 |  | 21.9 | 28.0 |  | 5.6 | 7.8 |  | 6.3 | 10.1 |  | 7.5 | 9.0 |  | 17.0 | 23.9 |  | 34.9 | 32.7 |  | 53.2 | 48.2 |  | 156.2 | 127.2 |
| [0 0 1] |  | 4.5 | 6.9 |  | 10.4 | 19.4 |  | 1.3 | 3.0 |  | 6.4 | 16.9 |  | 0.0 | 0.0 |  | 2.3 | 5.1 |  | 5.8 | 6.3 |  | 5.5 | 7.1 |  | 36.2 | 33.0 |
| [1 1 0] |  | 1.3 | 2.0 |  | 5.2 | 8.2 |  | 0.5 | 1.4 |  | 0.1 | 0.3 |  | 0.0 | 0.0 |  | 0.1 | 0.3 |  | 2.2 | 4.5 |  | 1.8 | 3.5 |  | 11.1 | 11.7 |
| [1 0 1] |  | 6.3 | 8.9 |  | 5.3 | 6.2 |  | 1.3 | 3.1 |  | 1.3 | 4.3 |  | 0.0 | 0.0 |  | 0.5 | 1.4 |  | 5.4 | 12.5 |  | 5.9 | 10.5 |  | 25.9 | 39.3 |
| [0 1 1] |  | 40.1 | 39.9 |  | 48.5 | 32.5 |  | 18.3 | 19.3 |  | 31.5 | 31.2 |  | 42.3 | 50.3 |  | 49.4 | 53.6 |  | 77.3 | 48.6 |  | 81.8 | 45.8 |  | 389.2 | 200.2 |
| [1 1 1] |  | 85.9 | 38.8 |  | 97.8 | 32.8 |  | 26.4 | 38.1 |  | 16.0 | 16.7 |  | 20.3 | 22.7 |  | 19.5 | 19.4 |  | 122.3 | 87.8 |  | 105.2 | 59.7 |  | 493.3 | 210.5 |
|  |  |  |  |  |  |  |  |  |  |  |  |  |  |  |  |  |  |  |  |  |  |  |  |  |  |  |  |
| *Sign. and non-significant profiles* | | | | | | |  |  |  |  |  |  |  |  |  |  |  |  |  |  |  |  |  |  |  |  |  |
| [1 0 0] |  | 45.3 | 30.5 |  | 35.3 | 27.9 |  | 8.3 | 15.3 |  | 4.4 | 6.1 |  | 1.0 | 3.5 |  | 0.8 | 1.1 |  | 23.0 | 43.6 |  | 11.9 | 16.0 |  | 129.9 | 88.8 |
| [0 1 0] |  | 21.8 | 18.5 |  | 38.1 | 30.4 |  | 7.0 | 7.7 |  | 8.5 | 13.7 |  | 12.3 | 13.4 |  | 19.3 | 24.5 |  | 59.2 | 39.3 |  | 84.4 | 58.2 |  | 250.4 | 154.5 |
| [0 0 1] |  | 16.9 | 13.5 |  | 22.1 | 25.3 |  | 8.2 | 12.6 |  | 11.2 | 21.6 |  | 3.8 | 7.3 |  | 8.3 | 12.3 |  | 30.0 | 19.7 |  | 21.6 | 16.5 |  | 121.9 | 62.6 |
| [1 1 0] |  | 14.6 | 17.3 |  | 20.7 | 16.1 |  | 1.6 | 2.6 |  | 0.9 | 2.2 |  | 0.8 | 2.3 |  | 1.0 | 1.9 |  | 13.4 | 18.1 |  | 10.6 | 8.8 |  | 63.5 | 37.2 |
| [1 0 1] |  | 13.6 | 11.8 |  | 15.3 | 7.4 |  | 4.6 | 7.9 |  | 3.0 | 9.5 |  | 0.8 | 2.6 |  | 2.6 | 4.1 |  | 16.5 | 19.7 |  | 19.6 | 18.7 |  | 75.8 | 58.8 |
| [0 1 1] |  | 58.3 | 42.4 |  | 72.4 | 44.3 |  | 28.6 | 27.6 |  | 35.9 | 35.3 |  | 51.6 | 51.5 |  | 57.1 | 54.1 |  | 115.2 | 56.1 |  | 107.8 | 50.7 |  | 526.8 | 205.5 |
|  |  |  |  |  |  |  |  |  |  |  |  |  |  |  |  |  |  |  |  |  |  |  |  |  |  |  |  |
| All |  | 170.3 | 49.3 |  | 203.8 | 44.8 |  | 58.3 | 44.3 |  | 63.9 | 71.1 |  | 70.1 | 56.9 |  | 88.9 | 47.9 |  | 257.3 | 95.9 |  | 255.8 | 97.1 |  | 1168 | 297.3 |

Table S2: Task-based (High-frequency) functional coupling (Z(r)) between voxels preferring the same task and voxels preferring different tasks. The upper panel of the table (Mono-preference voxels with significant profile) is depicted in Figure 4A-B.

|  |  |  |  | Within ROI (Distance matched) ^#^ | | | | | | |  | Between ROIs ^##^ | | | | | | |
| --- | --- | --- | --- | --- | --- | --- | --- | --- | --- | --- | --- | --- | --- | --- | --- | --- | --- | --- |
|  |  | Conditions |  | Descriptive statistics | | |  | Group analysis^§^ | | |  | Descriptive statistics | | |  | Group analysis^§^ | | |
|  |  |  |  | n | M | SD |  | t | df | p 1-t |  | n | M | SD |  | t | df | p 1-t |
| *Mono-preference voxels with significant task profile.* | | | |  | | | | | | |  |  | | | | | | |
|  | All 3 tasks | Same preference voxels |  | 12 | 1.815 | 0.586 |  | **3.7 | 11 | 0.0018 |  | 12 | 1.540 | 0.596 |  | **4.7 | 11 | 0.0003 |
|  |  | Different preference voxels |  | 12 | 1.762 | 0.590 |  |  |  |  |  | 12 | 1.454 | 0.594 |  |  |  |  |
|  | Erikson | Same preference voxels |  | 12 | 1.796 | 0.613 |  | **3.1 | 11 | 0.0052 |  | 12 | 1.507 | 0.661 |  | **4.1 | 11 | 0.0008 |
|  |  | Different preference voxels |  | 12 | 1.737 | 0.619 |  |  |  |  |  | 12 | 1.433 | 0.617 |  |  |  |  |
|  | Backmatching | Same preference voxels |  | 12 | 1.820 | 0.576 |  | **3.8 | 11 | 0.0014 |  | 12 | 1.557 | 0.593 |  | **5.0 | 11 | 0.0002 |
|  |  | Different preference voxels |  | 12 | 1.771 | 0.583 |  |  |  |  |  | 12 | 1.462 | 0.592 |  |  |  |  |
|  | Switching | Same preference voxels |  | 12 | 1.828 | 0.577 |  | **3.4 | 11 | 0.0028 |  | 12 | 1.555 | 0.594 |  | **4.5 | 11 | 0.0005 |
|  |  | Different preference voxels |  | 12 | 1.778 | 0.579 |  |  |  |  |  | 12 | 1.468 | 0.584 |  |  |  |  |
|  |  |  |  |  |  |  |  |  |  |  |  |  |  |  |  |  |  |  |
|  | *Extension to all preferences classes and all task profiles* | | | |  |  |  |  |  |  |  |  |  |  |  |  |  |  |
|  | Mono-preference – Sign. and non-sign. task profiles. | | |  |  |  |  |  |  |  |  |  |  |  |  |  |  |  |
|  | All 3 tasks | Same preference voxels |  | 12 | 1.775 | 0.577 |  | 1.0 | 11 | 0.1587 |  | 12 | 1532. | 0.617 |  | **3.6 | 11 | 0.0021 |
|  |  | Different preference voxels |  | 12 | 1.764 | 0.594 |  |  |  |  |  | 12 | 1.475 | 0.614 |  |  |  |  |
|  | All preference types – Significant task profiles only. | | |  |  |  |  |  |  |  |  |  |  |  |  |  |  |  |
|  | All 3 tasks | Same preference voxels |  | 12 | 1.890 | 0.554 |  | **3.2 | 11 | 0.0043 |  | 12 | 1.515 | 0.604 |  | **2.8 | 11 | 0.0093 |
|  |  | Different preference voxels |  | 12 | 1.858 | 0.573 |  |  |  |  |  | 12 | 1.498 | 0.608 |  |  |  |  |
|  | All preference types – Sign. and non-sign. task profiles. | | | |  |  |  |  |  |  |  |  |  |  |  |  |  |  |
|  | All 3 tasks | Same preference voxels |  | 12 | 1.859 | 0.556 |  | *2.0 | 11 | 0.0338 |  | 12 | 1.516 | 0.608 |  | *2.3 | 11 | 0.0223 |
|  |  | Different preference voxels |  | 12 | 1.842 | 0.574 |  |  |  |  |  | 12 | 1.502 | 0.613 |  |  |  |  |

Note. # Analysis confined to voxel pairs with both elements in the same ROI, matched for Euclidean distance. ## Analysis confined to voxel pairs with each element in a different ROI. § For each participant the average correlation in each condition was entered into a repeated measures t-test. Probabilities are one-tailed: * significant at 0.05; ** significant at Bonferroni corrected significance level to compensate for multiple comparisons.

Table S3: High-frequency (task-based) functional coupling (Z(r)) between voxels with the same task profile, during execution of the preferred task(s) and the non-preferred task(s). The upper table panel (Mono-preference voxels with significant task profile) is depicted in Figure 4C-D.

|  |  |  |  | Within ROI (Distance matched) ^#^ | | | | | | |  | Between ROIs ^##^ | | | | | | |
| --- | --- | --- | --- | --- | --- | --- | --- | --- | --- | --- | --- | --- | --- | --- | --- | --- | --- | --- |
|  |  | Conditions |  | Descriptive statistics | | |  | Group analysis^§^ | | |  | Descriptive statistics | | |  | Group analysis^§^ | | |
|  |  |  |  | n | M | SD |  | t | df | p 1-t |  | n | M | SD |  | t | df | p 1-t |
| *Mono-preference voxels with significant task profile.* | | | |  | | | | | | |  |  | | | | | | |
|  | All 3 profiles | During preferred task |  | 12 | 1.749 | 0.604 |  | 0.1 | 11 | 0.4657 |  | 12 | 1.561 | 0.611 |  | 1.1 | 11 | 0.1544 |
|  |  | During non-preferred tasks |  | 12 | 1.747 | 0.592 |  |  |  |  |  | 12 | 1.529 | 0.591 |  |  |  |  |
|  | [1 0 0] | During preferred task |  | 12 | 1.606 | 0.608 |  | -0.8 | 11 | 0.7863 |  | 11 | 1.358 | 0.623 |  | -0.5 | 10 | 0.6977 |
|  |  | During non-preferred tasks |  | 12 | 1.631 | 0.573 |  |  |  |  |  | 11 | 1.378 | 0.567 |  |  |  |  |
|  | [0 1 0] | During preferred task |  | 12 | 1.822 | 0.553 |  | 0.2 | 11 | 0.4301 |  | 12 | 1.591 | 0.570 |  | 0.6 | 11 | 0.2774 |
|  |  | During non-preferred tasks |  | 12 | 1.818 | 0.582 |  |  |  |  |  | 12 | 1.572 | 0.582 |  |  |  |  |
|  | [0 0 1] | During preferred task |  | 11 | 1.669 | 0.514 |  | 1.2 | 10 | 0.1375 |  | 11 | 1.420 | 0.547 |  | 1.0 | 10 | 0.1700 |
|  |  | During non-preferred tasks |  | 11 | 1.629 | 0.560 |  |  |  |  |  | 11 | 1.392 | 0.561 |  |  |  |  |
|  |  |  |  |  |  |  |  |  |  |  |  |  |  |  |  |  |  |  |
|  | *Extension to all preferences classes and all task profiles* | | | |  |  |  |  |  |  |  |  |  |  |  |  |  |  |
|  | Mono-preference – Sign. and non-sign. task profiles. | | |  |  |  |  |  |  |  |  |  |  |  |  |  |  |  |
|  | All 3 profiles | During preferred task |  | 12 | 1.719 | 0.607 |  | 0.1 | 11 | 0.4583 |  | 12 | 1.540 | 0.624 |  | 0.7 | 11 | 0.2453 |
|  |  | During non-preferred tasks |  | 12 | 1.717 | 0.592 |  |  |  |  |  | 12 | 1.528 | 0.615 |  |  |  |  |
|  | All preference types – Significant task profiles only. | | |  |  |  |  |  |  |  |  |  |  |  |  |  |  |  |
|  | All 6 profiles | During preferred task |  | 12 | 1.784 | 0.564 |  | 1.6 | 11 | 0.0701 |  | 12 | 1.528 | 0.598 |  | 1.5 | 11 | 0.0837 |
|  |  | During non-preferred tasks |  | 12 | 1.745 | 0.584 |  |  |  |  |  | 12 | 1.488 | 0.625 |  |  |  |  |
|  | All preference types – Sign. and non-sign. task profiles. | | | |  |  |  |  |  |  |  |  |  |  |  |  |  |  |
|  | All 6 profiles | During preferred task |  | 12 | 1.759 | 0.564 |  | 1.6 | 11 | 0.0678 |  | 12 | 1.529 | 0.600 |  | 1.4 | 11 | 0.0975 |
|  |  | During non-preferred tasks |  | 12 | 1.726 | 0.586 |  |  |  |  |  | 12 | 1.495 | 0.627 |  |  |  |  |

Note. # Analysis confined to voxel pairs with both elements in the same ROI, matched for Euclidean distance. ## Analysis confined to voxel pairs with each element in a different ROI. § For each participant the average correlation in each condition was entered into a repeated measures t-test. Probabilities are one-tailed: * significant at 0.05; ** significant at Bonferroni corrected significance level to compensate for multiple comparisons.

Table S4: Low-frequency (resting state) functional coupling (Z(r)) between voxels preferring the same task and voxels preferring different tasks. The upper panel of the table (Mono-preference voxels with significant task profile) is depicted in Figure 5A-B.

|  |  |  |  | Within ROI (Distance matched) ^#^ | | | | | | |  | Between ROIs ^##^ | | | | | | |
| --- | --- | --- | --- | --- | --- | --- | --- | --- | --- | --- | --- | --- | --- | --- | --- | --- | --- | --- |
|  |  | Conditions |  | Descriptive statistics | | |  | Group analysis^§^ | | |  | Descriptive statistics | | |  | Group analysis^§^ | | |
|  |  |  |  | n | M | SD |  | t | df | p 1-t |  | n | M | SD |  | t | df | p 1-t |
| *Mono-preference voxels with significant task profile.* | | | |  | | | | | | |  |  | | | | | | |
|  | All 3 profiles | Same preference voxels |  | 12 | 0.426 | 0.195 |  | **3.6 | 11 | 0.0019 |  | 12 | 0.171 | 0.065 |  | **7.6 | 11 | 0.0000 |
|  |  | Different preference voxels |  | 12 | 0.353 | 0.226 |  |  |  |  |  | 12 | 0.110 | 0.067 |  |  |  |  |
|  | [1 0 0] | Same preference voxels |  | 12 | 0.479 | 0.267 |  | *2.2 | 11 | 0.0270 |  | 11 | 0.141 | 0.101 |  | 1.5 | 10 | 0.0831 |
|  |  | Different preference voxels |  | 12 | 0.424 | 0.254 |  |  |  |  |  | 11 | 0.094 | 0.076 |  |  |  |  |
|  | [0 1 0] | Same preference voxels |  | 12 | 0.387 | 0.102 |  | 0.9 | 11 | 0.1847 |  | 12 | 0.141 | 0.062 |  | **2.7 | 11 | 0.0108 |
|  |  | Different preference voxels |  | 12 | 0.348 | 0.221 |  |  |  |  |  | 12 | 0.106 | 0.066 |  |  |  |  |
|  | [0 0 1] | Same preference voxels |  | 11 | 0.699 | 0.282 |  | **4.6 | 10 | 0.0005 |  | 11 | 0.238 | 0.146 |  | 1.8 | 10 | 0.0523 |
|  |  | Different preference voxels |  | 11 | 0.557 | 0.247 |  |  |  |  |  | 11 | 0.148 | 0.087 |  |  |  |  |
|  |  |  |  |  |  |  |  |  |  |  |  |  |  |  |  |  |  |  |
|  | *Extension to all preferences classes and all task profiles* | | | |  |  |  |  |  |  |  |  |  |  |  |  |  |  |
|  | Mono-preference – Sign. and non-sign. task profiles. | | |  |  |  |  |  |  |  |  |  |  |  |  |  |  |  |
|  | All 3 profiles | Same preference voxels |  | 12 | 0.327 | 0.112 |  | **6.2 | 11 | 0.0000 |  | 12 | 0.125 | 0.064 |  | **9.7 | 11 | 0.0000 |
|  |  | Different preference voxels |  | 12 | 0.268 | 0.135 |  |  |  |  |  | 12 | 0.085 | 0.063 |  |  |  |  |
|  | All preference types – Significant task profiles only. | | |  |  |  |  |  |  |  |  |  |  |  |  |  |  |  |
|  | All 6 profiles | Same preference voxels |  | 12 | 0.487 | 0.151 |  | **5.1 | 11 | 0.0002 |  | 12 | 0.168 | 0.056 |  | **4.9 | 11 | 0.0003 |
|  |  | Different preference voxels |  | 12 | 0.433 | 0.148 |  |  |  |  |  | 12 | 0.139 | 0.054 |  |  |  |  |
|  | All preference types – Sign. and non-sign. task profiles. | | | |  |  |  |  |  |  |  |  |  |  |  |  |  |  |
|  | All 6 profiles | Same preference voxels |  | 12 | 0.405 | 0.117 |  | **8.7 | 11 | 0.0000 |  | 12 | 0.135 | 0.059 |  | **6.8 | 11 | 0.0000 |
|  |  | Different preference voxels |  | 12 | 0.339 | 0.109 |  |  |  |  |  | 12 | 0.097 | 0.059 |  |  |  |  |

Note. # Analysis confined to voxel pairs with both elements in the same ROI, matched for Euclidean distance. ## Analysis confined to voxel pairs with each element in a different ROI. § For each participant the average correlation in each condition was entered into a repeated measures t-test. Probabilities are one-tailed: * significant at 0.05; ** significant at Bonferroni corrected significance level to compensate for multiple comparisons.

Table S5: Low-frequency (resting state) functional connectivity profile similarity (ε²) between voxels preferring the same task and voxels preferring different tasks. The upper panel of the table (Mono-preference voxels with significant task profile) is depicted in Figure 5C-D.

|  |  |  |  | Within ROI (Distance matched) ^#^ | | | | | | |  | Between ROIs ^##^ | | | | | | |
| --- | --- | --- | --- | --- | --- | --- | --- | --- | --- | --- | --- | --- | --- | --- | --- | --- | --- | --- |
|  |  | Conditions |  | Descriptive statistics | | |  | Group analysis^§^ | | |  | Descriptive statistics | | |  | Group analysis^§^ | | |
|  |  |  |  | n | M | SD |  | t | df | p 1-t |  | n | M | SD |  | t | df | p 1-t |
| *Mono-preference voxels with significant task profile.* | | | |  | | | | | | |  |  | | | | | | |
|  | All 3 profiles | Same preference voxels |  | 12 | 0.728 | 0.075 |  | *2.0 | 11 | 0.0369 |  | 12 | 0.635 | 0.056 |  | **3.4 | 11 | 0.0031 |
|  |  | Different preference voxels |  | 12 | 0.693 | 0.120 |  |  |  |  |  | 12 | 0.599 | 0.057 |  |  |  |  |
|  | [1 0 0] | Same preference voxels |  | 12 | 0.724 | 0.110 |  | 1.1 | 11 | 0.1454 |  | 11 | 0.612 | 0.090 |  | 0.6 | 10 | 0.2659 |
|  |  | Different preference voxels |  | 12 | 0.703 | 0.135 |  |  |  |  |  | 11 | 0.585 | 0.071 |  |  |  |  |
|  | [0 1 0] | Same preference voxels |  | 12 | 0.732 | 0.051 |  | 1.2 | 11 | 0.1327 |  | 12 | 0.609 | 0.057 |  | 1.1 | 11 | 0.1439 |
|  |  | Different preference voxels |  | 12 | 0.702 | 0.120 |  |  |  |  |  | 12 | 0.596 | 0.058 |  |  |  |  |
|  | [0 0 1] | Same preference voxels |  | 11 | 0.820 | 0.098 |  | **2.9 | 10 | 0.0073 |  | 11 | 0.685 | 0.089 |  | 1.8 | 10 | 0.0514 |
|  |  | Different preference voxels |  | 11 | 0.773 | 0.095 |  |  |  |  |  | 11 | 0.629 | 0.064 |  |  |  |  |
|  |  |  |  |  |  |  |  |  |  |  |  |  |  |  |  |  |  |  |
|  | *Extension to all preferences classes and all task profiles* | | | |  |  |  |  |  |  |  |  |  |  |  |  |  |  |
|  | Mono-preference – Sign. and non-sign. task profiles. | | |  |  |  |  |  |  |  |  |  |  |  |  |  |  |  |
|  | All 3 profiles | Same preference voxels |  | 12 | 0.688 | 0.053 |  | **3.5 | 11 | 0.0026 |  | 12 | 0.606 | 0.047 |  | **4.8 | 11 | 0.0003 |
|  |  | Different preference voxels |  | 12 | 0.652 | 0.082 |  |  |  |  |  | 12 | 0.579 | 0.057 |  |  |  |  |
|  | All preference types – Significant task profiles only. | | |  |  |  |  |  |  |  |  |  |  |  |  |  |  |  |
|  | All 6 profiles | Same preference voxels |  | 12 | 0.752 | 0.060 |  | **4.8 | 11 | 0.0003 |  | 12 | 0.636 | 0.050 |  | **4.0 | 11 | 0.0011 |
|  |  | Different preference voxels |  | 12 | 0.727 | 0.061 |  |  |  |  |  | 12 | 0.618 | 0.051 |  |  |  |  |
|  | All preference types – Sign. and non-sign. task profiles. | | | |  |  |  |  |  |  |  |  |  |  |  |  |  |  |
|  | All 6 profiles | Same preference voxels |  | 12 | 0.701 | 0.052 |  | **7.9 | 11 | 0.0000 |  | 12 | 0.608 | 0.048 |  | **4.3 | 11 | 0.0006 |
|  |  | Different preference voxels |  | 12 | 0.673 | 0.059 |  |  |  |  |  | 12 | 0.583 | 0.053 |  |  |  |  |

Note. # Analysis confined to voxel pairs with both elements in the same ROI, matched for Euclidean distance. ## Analysis confined to voxel pairs with each element in a different ROI. § For each participant the average correlation in each condition was entered into a repeated measures t-test. Probabilities are one-tailed: * significant at 0.05; ** significant at Bonferroni corrected significance level to compensate for multiple comparisons.

Table S6: Low-frequency (resting state) functional connectivity strength (Z(r)) between voxels preferring the same task and voxels preferring different tasks for the two participants whose resting state data were collected prior to entering the study.

|  |  |  | |  | | Within ROI (Distance matched) ^#^ | | | | | | | | | | |  | | | | | Between ROIs ^##^ | | | | | | | | | | | | | | | | | | | | | | | | | | |  |  |  |  |  |  |
| --- | --- | --- | --- | --- | --- | --- | --- | --- | --- | --- | --- | --- | --- | --- | --- | --- | --- | --- | --- | --- | --- | --- | --- | --- | --- | --- | --- | --- | --- | --- | --- | --- | --- | --- | --- | --- | --- | --- | --- | --- | --- | --- | --- | --- | --- | --- | --- | --- | --- | --- | --- | --- | --- | --- |
|  |  | Conditions | |  | | Descriptive statistics | | |  | | Group analysis^§^ | | | | | |  | | | | | Descriptive statistics | | | | | | | | |  | | | Group analysis^§^ | | | | | | | | | | | | | | |  |  |  |  |  |  |
|  |  |  | |  | | n | M | SD |  | | t |  | | | | p 1-t |  | | | | | n | | M | | | SD | | | |  | | | t | | | | | df | | | | | | p 1-t | | | |  |  |  |  |  |  |
|  | *Participant 08:* | | | | | |  |  |  | |  |  | | | |  |  | | | | |  | |  | | |  | | | | | | | |  | | |  | | | | |  | | | | |  | | | | | |  |
|  | Mono-preference – Significant task profiles only. | | | | |  |  |  |  | |  |  | | | |  |  | | | | |  | |  | | | | | |  | | | | |  | | |  | | | | |  | | | | |  | | | | |  |  |
|  | All 3 profiles | Same preference voxels |  | | 1285 | | 0.248 | 0.265 |  | | 114.9 |  | | <0.0001 | | |  | | | | | 24908 | | | 0.126 | | | 0.187 | | | |  | | | 313.6 | | | | | |  | | | <0.0001 | | | | | |  |  |  |  |  |
|  |  | Different preference voxels |  | | 1285 | | 0.121 | 0.061 |  | |  |  | | | |  |  | | | | | 15990 | | | 0.092 | | | 0.194 | | | |  | | | | |  | | |  | | | | | | |  | | | |  |  |  |  |
|  | Mono-preference – Sign. and non-sign. task profiles. | | | | |  |  |  |  | |  |  | | | |  |  | | | | |  | |  | | | | | |  | | | | |  | | |  | | | | |  | | | | |  | | | | |  |  |
|  | All 3 profiles | Same preference voxels |  | | 7167 | | 0.288 | 0.300 |  | 365.6 | | |  | | <0.0001 | | |  | | | | | 51194 | | | 0.120 | | | 0.181 | | | |  | | | 341.8 | | | | | |  | | | | <0.0001 | | | | | |  |  |  |
|  |  | Different preference voxels |  | | 7167 | | 0.191 | 0.309 |  | |  |  | | | |  |  | | | | | 66674 | | | 0.100 | | | 0.182 | | | |  | | | | |  | | |  | | | | | | |  | | | |  |  |  |  |
|  | All preference types – Significant task profiles only. | | | | |  |  |  |  | |  |  | | | |  |  | | | | |  | |  | | | | | |  | | | | |  | | |  | | | | |  | | | | |  | | | | |  |  |
|  | All 6 profiles | Same preference voxels |  | | 5526 | | 0.396 | 0.356 |  | 269.6 | | |  | | <0.0001 | | |  | | | | | 27848 | | | 0.126 | | | 0.188 | | | |  | | | 137.3 | | | | | |  | | | | <0.0001 | | | | | |  |  |  |
|  |  | Different preference voxels |  | | 5526 | | 0.281 | 0.377 |  | |  |  | | | |  |  | | | | | 54705 | | | 0.109 | | | 0.194 | | | |  | | | | |  | | |  | | | | | | |  | | | |  |  |  |  |
|  | All preference types – Sign. and non-sign. task profiles. | | | | | |  |  |  | |  |  | | | |  |  | | | | |  | |  | | |  | | | | | | | |  | | |  | | | | |  | | | | |  | | | | | |  |
|  | All 6 profiles | Same preference voxels |  | | 1667 | | 0.381 | 0.364 |  | 358.0 | | |  | | <0.0001 | | |  | | | | | 63584 | | | 0.608 | | | 0.048 | | | |  | | | 195.7 | | | | | |  | | | | <0.0001 | | | | | |  |  |  |
|  |  | Different preference voxels |  | | 1667 | | 0.305 | 0.373 |  | |  |  | | | |  |  | | | | 210483 | | | | 0.105 | | | 0.181 | | | |  | | | | |  | | |  | | | | | | |  | | | |  |  |  |  |
|  | *Participant 11:* | | | | | |  |  |  | |  |  | | | |  |  | | | | |  | |  | | |  | | | | | | | |  | | |  | | | | |  | | | | |  | | | | | |  |
|  | Mono-preference – Significant task profiles only. | | | | |  |  |  |  | |  |  | | | |  |  | | | | |  | |  | | | | | |  | | | | |  | | |  | | | | |  | | | | |  | | | | |  |  |
|  | All 3 profiles | Same preference voxels |  | | 995 | | 0.269 | 0.302 |  | 253.1 | | |  | | <0.0001 | | |  | | | | | 61358 | | | 0.101 | | | 0.150 | | | |  | | | 2559.6 | | | | | |  | | | | <0.0001 | | | | | |  |  |  |
|  |  | Different preference voxels |  | | 995 | | 0.066 | 0.266 |  | |  |  | | | |  |  | | | | | 16600 | | | 0.036 | | | 0.135 | | | |  | | | | |  | | |  | | | | | | |  | | | |  |  |  |  |
|  | Mono-preference – Sign. and non-sign. task profiles. | | | | |  |  |  |  | |  |  | | | |  |  | | | | |  | |  | | | | | |  | | | | |  | | |  | | | | |  | | | | |  | | | | | |  |
|  | All 3 profiles | Same preference voxels |  | | 12567 | | 0.168 | 0.250 |  | 461.8 | | |  | | <0.0001 | | |  | | 140853 | | | | | | 0.076 | | | 0.146 | | | |  | | | 10775 | | | | | |  | | | | <0.0001 | | | | | |  |  |  |
|  |  | Different preference voxels |  | | 12567 | | 0.105 | 0.217 |  | |  |  | | | |  |  | | 117500 | | | | | | 0.020 | | | 0.126 | | | |  | | | | |  | | |  | | | | | | |  | | | | | | | |

(Table S2 – continued)

|  | All preference types – Significant task profiles only. | | | |  |  |  |  | |  |  | | |  |  | | | |  |  | | |  | | |  | | |  | | |  | | |  | | |  |  |
| --- | --- | --- | --- | --- | --- | --- | --- | --- | --- | --- | --- | --- | --- | --- | --- | --- | --- | --- | --- | --- | --- | --- | --- | --- | --- | --- | --- | --- | --- | --- | --- | --- | --- | --- | --- | --- | --- | --- | --- |
|  | All 6 profiles | Same preference voxels |  | 17133 | | 0.268 | 0.309 |  | 20.3 | | |  | <0.0001 | | |  | | 107566 | | | | 0.094 | | | 0.156 | | |  | | 1695.5 | | | |  | | <0.0001 | | | |
|  |  | Different preference voxels |  | 17133 | | 0.253 | 0.310 |  | |  |  | | |  |  | | 181590 | | | | 0.069 | | | 0.153 | | |  | | | |  | |  | | | |  | |  |
|  | All preference types – Sign. and non-sign. task profiles. | | | | |  |  |  | |  |  | | |  |  | | | |  |  | | |  | | |  | | |  | | |  | | |  | | |  |  |
|  | All 6 profiles | Same preference voxels |  | 51734 | | 0.228 | 0.309 |  | 455.0 | | |  | <0.0001 | | |  | | 256014 | | | | 0.073 | | | 0.147 | | |  | | 10905 | | | |  | | <0.0001 | | | |
|  |  | Different preference voxels |  | 51734 | | 0.188 | 0.295 |  | |  |  | | |  |  | | 688407 | | | | 0.039 | | | 0.139 | | |  | | | |  | |  | | | |  | |  |

Note. # Analysis confined to voxel pairs with both elements in the same ROI, matched for Euclidean distance. ## Analysis confined to voxel pairs with each element in a different ROI.
